# Explainable AI Model Reveals Informative Mutational Signatures for Cancer-Type Classification

**DOI:** 10.3390/cancers17111731

**Published:** 2025-05-22

**Authors:** Jonas Wagner, Jan Oldenburg, Neetika Nath, Stefan Simm

**Affiliations:** 1 Institute of Bioinformatics, University Medicine Greifswald, 17475 Greifswald, Germany; jonas.wagner@uni-greifswald.de (J.W.); jan.oldenburg@uni-greifswald.de (J.O.);; 2 Institute of Bioanalysis, Department of Applied Sciences, Coburg University of Applied Sciences and Arts, 96450 Coburg, Germany

**Keywords:** XAI, mutational signatures, informative mutational signatures, cancer types, driver genes, whole genome sequencing

## Abstract

**Background/Objectives**: The prediction of cancer types is primarily reliant on driver genes and their specific mutations. The advancement in novel omics technologies has led to the acquisition of additional genetic data. When integrated with artificial intelligence models, there is considerable potential for this to enhance the accuracy of cancer diagnosis. As mutational signatures can provide insights into repair mechanism malfunctions, they also have the potential for more accurate cancer diagnosis. **Methods**: First, we compared unsupervised and supervised machine learning approaches to predict cancer types. We employed deep and artificial neural network architectures with an explainable component like layerwise relevance propagation to extract the most relevant features for the cancer-type prediction. Ten-fold cross-validation and an extensive grid search were used to optimize the neural network architecture using driver gene mutations, mutational signatures and topological mutation information as input. The PCAWG dataset was used as input to discriminate between 17 primary sites and 24 cancer types. **Results**: Overall, our approach showed that the most relevant mutation information to discriminate between cancer types is increased by >10% using the whole genome or intergenic and intronic genome regions instead of exome information. Furthermore, the most relevant features for most cancer types, except for two, are in the mutational signatures and not the topological mutation information. **Conclusions**: Informative mutational signatures outperformed the prediction of cancer types in comparison to driver gene mutations and added a new layer of diagnostic information. As the degree of information within the mutational signatures is not solely based on the frequency of occurrence, it is even possible to separate cancer types from the same primary site by the different relevant mutations. Furthermore, the comparison of informative mutational signatures allowed the cancer-type assignment of specific impaired repair mechanisms.

## Simple Summary

The objective of this research is to enhance the prediction of cancer types using an explainable artificial intelligence (XAI) model based on an artificial neural network with layerwise relevance propagation to extract informative mutational signatures. Multiple XAI models have been optimized using 10-fold cross-validation and grid search. In contrast to earlier approaches, the study compares the prediction capacities of unsupervised and supervised approaches. As outcomes, the paper showed better cancer-type-prediction accuracies using whole genome or intronic/intergenic mutation information instead of exome regions alone. Furthermore, the usage of mutational signatures is more relevant for prediction than localization information or driver gene mutation information. Overall, the XAI models developed in this study enabled informative mutational signatures to be generated for cancer-type and primary-site classification, leading to the detection of differences in the mechanistic characteristics of cancer types. These informative mutational signatures can be used in the future to more accurately and robustly diagnose cancer types as well as a foundation from which to identify new potential biomarkers and their context of impaired repair mechanisms.

## 1. Introduction

Modern clinical approaches in cancer diagnostics are based on histological and anatomic data to determine the tumor’s histological type. Other ways of identifying the site of origin are immunohistochemistry (IHC) [1]. Both techniques of diagnostic pathology need manual interpretation that can lead to false positive results [2]. To support these methods as well as increase the prediction accuracy, nowadays, high-throughput approaches like NGS are used to allow better differentiation [3]. These NGS approaches can be based on mRNAs or miRNAs [4] as well as DNA methylation pattern [5]. Nowadays, whole genome sequencing (WGS) and whole exome sequencing (WES) technologies in the clinical environment enable the accurate identification of diseases based on specific mutation markers and driver genes [6]. Four evolutionary forces affect allele frequencies in populations of individuals and cells, namely mutagenesis, natural selection, genetic drift, and gene flow [7]. In general, mutations can be discriminated in by the base substitutions of single nucleotides, insertions or deletions of short DNA segments (InDels), rearrangements of longer DNA segments within a chromosome as well as copy number increases or reductions in chromosomes [8]. For single nucleotide changes, we can roughly discriminate between variations (SNVs) and polymorphisms (SNPs) [9]. SNPs are, in contrast to SNVs, present in at least 1% of the analyzed population [10].

In the medical context, the local and global analysis of SNPs and SNVs can be used to diagnose specific diseases like congenital heart disease [11] as well as possible risk factors for diseases like venous thrombosis [12]. Thereby, SNVs and SNPs are mostly related to specific driver genes and biomarkers to detect specific diseases like breast cancer [13]. Such markers can identify differences in susceptibility and allow the targeted treatment of cancer types [14]. Besides these few driver mutations, in the past, most other mutations have been assigned as passenger mutations and treated like site information [15,16]. Recently, genomic instability has been identified as a hallmark of cancer diagnosis related to impaired repair mechanisms [17]. Large-scale sequencing and genomic characterization efforts have contributed to the generation of so-called mutational signatures to identify phenotypic consequences within sequencing data [15]. These mutational signatures are characteristic patterns that can be used to obtain insights into DNA damage caused by endogenous and exogenous mutagens, as well as the affected DNA repair and copy mechanisms [18]. One source for such signatures is the COSMIC database, currently containing 67 different single-base substitution (SBS) signatures [19], in close collaboration with tools like SigProfiler [20] or MuSiCal [21]. The SBS signatures describe the frequencies of mutation counts in a flanking nucleotide context (one-, two-, trinucleotide context). These patterns within the mutational signatures so far have been used to compare them against whole genome datasets of cancer patients. Comparisons of such SBSs with studies like the pan cancer analysis of whole genomes (PCAWG; [22]) enables the identification of similarities within specific cancer types. One problem soar is the mixture of patterns based on multiple impaired repair mechanisms and other environmental factors like smoking. Furthermore, the separation of different cancer types or subtypes in the same primary site is difficult, having an overall similar mutation frequency.

In recent years artificial intelligence (AI) approaches, including machine learning (ML) and deep learning (DL), have increasingly been used in imaging classification of cancers [23,24,25], reaching nearly perfect prediction accuracy (>99%) for specific cancer types [26]. Besides image information, NGS expression datasets have also become a source for predicting cancer types using artificial neural networks (ANN) [27] and CNNs [28], reaching over 90% accuracy differentiating 16–33 cancer types (Table 1). The main problem of NGS expression datasets is the fluctuation in expression pattern, influenced by cell type and individuals, making it hard for cancer subtyping [29]. To overcome this problem, the aim is to analyze the source of cancer diseases, meaning the accumulation of mutations and genetic alterations [30]. Training random forests (RFs), decision trees or Gaussian naïve Bayes on driver genes and their mutations associated with breast adenocarcinoma, they were able to reach 0.99 accuracy [31]. In contrast, the discrimination of many cancer types or even subtypes is much more complex. Studies using support vector machines (SVMs) on somatic mutation patterns (17 tumor sites) reached F1 scores of more than 0.7 for five of the cancer types [32]. RF classifiers on mutational signatures were able to identify the primary sites of cancers, with a classification accuracy of 0.85 [33]. Deep NNs (DNNs), trained on single somatic point mutations, reached an overall accuracy of 0.64 distinguishing between 12 cancer types [34]. In 2020, the usage of an ANN classifier outperformed this DNN [34] reaching an overall accuracy of 91% in the classification of 24 cancer types by adding topological mutation information [35] (Table 1).

Currently, most machine learning (ML) methods focus only on high accuracy values and work like a black-box, while often disregarding the relevant information for the decision of the ML-model prediction. As the approaches are well-suited to tackling complex problems, they generate individualized insights that often surpass the capabilities of traditional statistical methods. However, the deployment of ML models in these domains necessitates robust validation and control measures that surpass the usage of a single evaluation metric. NN are particularly vulnerable to challenges such as the “curse of dimensionality” [41,42] and the development of “Clever Hans predictors” [43]. At their core, NN models are optimization algorithms designed to minimize errors defined by a loss function. However, when spurious confounders strongly correlate with the labels, models can inadvertently rely on these confounders. Such models can achieve deceptively high-performance metrics, such as accuracy or F1 score, despite lacking generalizable predictive power.

In this study, we focus on the most relevant information within the somatic mutation signatures, driver gene mutations and topological mutation information to perform an accurate cancer-type prediction. To extract which of the proposed mutational information (topological, driver genes, mutational signatures) have the highest relevance to predict cancer types and subtypes, explainable AI (XAI) methods can help to understand the underlying concept of the NN decision. The examination of individual predictions provides insights into the features that drive model decisions. This allowed us to add a new informative layer into the mutational signatures, leading to the generation of informative mutational signatures. Overall, we could show that especially specific somatic mutations are the most relevant to discriminate specific cancer types that are not only based on occurrence or location. Additionally, the whole genome or even intergenic and intronic genome regions showed >10% higher accuracy in cancer-type prediction than solely the exome information. The informative mutational signatures give a more biologically informed approach assigning them to dysfunctions in DNA repair mechanisms. In the end, the informative mutational signatures allowed the prediction of specific cancer types in the same primary site. At first, we used unsupervised approaches, like PCA and clustering, to set the ground truth to differentiate cancer types, as well as to look for possible confounders within the PCAWG dataset. For the supervised learning, we performed a 10-fold cross-validation and grid search based on the ANN architecture used in Jiao et al. [35]. We used this ANN model as it showed very high accuracy in cancer-type prediction on somatic alteration information. Our approach extended their analysis to somatic alterations in only exonic, intronic, intergenic and whole genome information. By applying explainable components to the ANN model, it was possible to extract the most relevant mutation types for specific cancer types. The generation of informative mutational signatures by combining the mutation frequency and mutation importance for each cancer type outperformed the usage of solely driver gene mutations. Additionally, we assigned informative mutational signatures to dysfunctions in the DNA repair mechanism and this allowed us to discriminate between cancer types from the same primary site.

## 2. Materials and Methods

### 2.1. Generating Datasets of Mutational Signatures, Driver Genes and Topological Mutation Information

For our analysis, we used the open access data provided by the ICGC Data Portal (https://dcc.icgc.org/, accessed 7 October 2021). The data include somatic mutations in the Variant Call Format (vcf) from 2780 PCAWG cancer patients. Of the 37 cancer types in the PCAWG, ten were excluded from the analysis because of their low patient numbers (<35 patients). Our analysis is based on 2592 samples for 24 types of cancer originating from 18 primary sites. The generation of our datasets was performed on the somatic single point mutations from the vcf-files. We performed the generation of mutational signatures and topological mutation information once for the whole genome (WGS), once only on the exome (WES) and once on all intergenic and intronic regions (WIIS).

The mutational signatures were generated using SigProfilerExtractor (version 1.1.25) [20] for the 3-nucleotide-, 2-nucleotide- and 1-nucleotide contexts. For the 3-nucleotide context, the position before and after the point mutation are extracted, while for the 2-nucleotide context, only the nucleotides before or after the point mutation are used. The 1-nucleotide context only contains the point mutation. In total, 150 features per patient (96 3-nucleotide-mutations, 48 2-nucleotide-mutations and 6 1-nucleotide-mutations; MS) were saved in one data frame.

Similarly to Jiao et al. [35], we cut the genome (irrespective of chromosome borders) in one Mega-base-long DNA pieces, ending up with 2896 bins for the whole human genome. For each bin, we counted the number of single point mutations and generated the topological information data frame (Bins). In addition, we extracted the 7845 known cancer driver genes from the DriverDBv4 [44] and counted the number of single point mutations in each gene to generate a data frame of driver gene mutations (GeneM).

### 2.2. Unsupervised Clustering Methods on Mutational Signatures

A principal component analysis (PCA, R v. 4.0.3; library prcomp) was performed using the 3-nucleotide context mutational signature count (z-score normalized) for the WGS, WES and WIIS datasets. Coloring the patients was based on cancer type, primary site, sex or age. Additionally, we performed a hierarchical (R v. 4.0.4; h clust) and k-means clustering (R v. 4.0.4; kmeans) with Euclidean distance measure also based on the 3-nucleotide context of the mutational signatures.

### 2.3. Neural Network Architecture for Cancer-Type Prediction

Different architectures and hyperparameter optimizations of ANNs were implemented in pytorch (version 1.8.0; Python version 3.9.4). The hyperparameter optimization of the ANN was based on the Jiao et. al. [35] to set our grid search space. We used the 10 different architectures to set learning rate (0.0001; 0.00025), L2-penalty (0.001; 0.011), dropout rate (1 × 10^−6^; 0.5), number of layers (1; 4), number of neurons per layer (630; 1024) and activation function (relu, softplus). For each, we performed a short pre-training with a 10-fold cross-validation and obtained the best results for the model: layers: 4, neurons per layer: 1024, activation: relu (rectified linear unit activation function), learning rate: 0.000195, dropout rate: 1 × 10^−6^, L2-penalty: 0.001. For the training, we fixed 50 epochs with an early stopping mechanism, which terminated if the validation accuracy stagnated for 10 epochs. In that case, the epoch of first stagnation was used as a trained model. As inputs, we used the datasets of Table 2.

### 2.4. Cross-Validation and Explainability of Neural Network Models

To allow a robust and general evaluation of the ANN performance, we performed a 10-fold cross validation. In the beginning, we split our dataset of patients into primary sites or cancer types. We divided each class separately into 10 pieces of equal size and used 9 of them for training and 1 for validation. Afterwards, we iterated through the pieces to have each of the ten pieces once for validation. To prevent imbalance in the datasets for the different cancer types and primary sites, we used a special weighting of the inputs based on the number of samples in the classes. The weight for a class was calculated by dividing the total number of samples by the number of samples in the class, resulting in higher weights for smaller classes. The splits in training and validation were based on labels so it could be easily transferred to all data frames (Bin + MS, MS, GeneM), irrespective of WGS, WES and WIIS to allow direct comparison of the model performance between these approaches (Table 2).

(1) $$R_{i\leftarrow j}^{\left(l,l+1\right)}=\frac{z_{ij}}{z_{j}+\varepsilon }\times R_{j}^{\left(l+1\right)},z_{j}\geq 0\frac{z_{ij}}{z_{j}-\varepsilon }\times R_{j}^{\left(l+1\right)},z_{j}<0$$

Equation (1): LRP-Epsilon. The LRP-Epsilon equation is an extension of the base LRP method that introduces a stabilization parameter to address small values and improve numerical stability. In this equation, *i* and *j* describe the positions of specific layers within a neural network, with *j* being the layer immediately following *i*. The output of a neuron in layer *i*, including the activation function, and the weight associated with the connection between a neuron in layer *i* and a neuron in layer *j*. The term represents the sum of all for layer *i* of a neuron and its bias term. The total relevance is computed as the sum of all incoming relevance scores.

For the explainable component, we evaluated the ANN models with the epsilon rule from layer-wise relevance propagation LRP [45], implemented via the Zennit package (https://github.com/chr5tphr/zennit, version 0.5.1, accessed on 9 January 2025). The LRP operates by backpropagating the predicted value, computed prior to the softmax activation, through the network using the weights and inputs of individual neurons. As a local post hoc explainability method, LRP assigns a relevance score for each feature, offering insights into the contribution of input features to the model’s decision for a specific prediction. We employed the LRP-epsilon variant (Equation (1)), which incorporates a stabilization parameter ϵ to mitigate the risk of producing unbounded relevance values. This approach accommodates positive and negative relevance scores, enabling a more nuanced representation of feature contributions in comparison to the gamma method, while avoiding dependency on hyperparameter adjustments required by the alpha-beta method. To facilitate comparisons across different models, all relevance scores were normalized by dividing by the total absolute relevance values for each prediction.

To rank the feature importance of the XAI-ANN model in a global assessment, we computed the median, mean, standard deviation, minimum and maximum relevance scores for each predicted cancer type from the validation sets of the cross-validation. The median value is used for the informative mutational signatures. Furthermore, after obtaining the importance per feature from the LRP, we used the median values of these relevance values to cluster the cancer types in a heatmap (ComplexHeatmaps v. 2.6.2; [46]). For the analysis of the WGS_GeneM dataset and quantitative nature of the cancer driver genes from the DriverDBv4 [44], we developed a quantitative-LRP analysis. We specifically analyzed the frequency of features that contribute to 80% of the predicted relevance. To identify these genes, we summed the local relevance scores in descending order of their absolute values until the cumulative sum reached 80% of the pre-softmax value, which is used as the initial relevance by the LRP algorithm. This threshold is based on the frequency of driver genes reported by Michael S. Lawrence et al. [47].

### 2.5. Statistical Analysis

To evaluate the performance of the ANN models, we calculated precision, recall, F1 score and the Matthews correlation coefficient (MCC). In the following equations, the abbreviations TP (true positive), FP (false positive), TN (true negative) and FN (false negative) are used to describe if the predicted label and true label are the same (Equations (2)–(5)).

Precision = TP/(TP + FP)(2)

Recall = TP/(TP + FN)(3)

F1 score = 2 × (Recall × Precision)/(Recall + Precision)(4)

(5) $$MCC=\frac{TP\times TN-FP\times FN}{\sqrt{\left(TP+FN\right)\times \left(TP+FP\right)\times \left(TN+FP\right)\times (TN+FN)}}$$

(6) $$\rho_{X,Y}=\frac{cov(X,Y)}{\sigma_{X}\sigma_{Y}}$$

Equations (2)–(6): Metrics used for statistical analysis. Precision (2) is a measurement that when looking at all samples assigned to a cancer type shows the proportion of correct assignments. Recall (3) describes the proportion of samples belonging to a cancer type that were assigned to this cancer type by the model. The F1 score (4) is the harmonic mean of recall and precision. The MCC score (5) is a measure to explain the difference in the predicted and actual values of a contingency table for a cancer type. The Pearson correlation coefficient (6) measures a linear relationship between two variables.

For the comparison of produced signatures with the catalogue of known signatures, we used the Pearson correlation coefficient (Equation (6)). Cov describes the covariance between two cancer signatures, while σ_X_ and σ_Y_ describe the standard deviations of the two signatures. Additionally, we computed the feature-focused distance between two signatures. This distance was determined by calculating the absolute difference between corresponding features. The individual differences were then summed and normalized to the range [0,1] by dividing by the maximum possible distance of 2. For the hypothesis testing of significance of single important mutations, we calculated the *p*-values for the Wilcoxon tests on comparison of the mean number of the 5 most important mutation types for each cancer type with the other cancer types grouped together and with each cancer type separately. The *p*-values for the pairwise comparisons were adjusted using the Holm–Bonferroni method.

### 2.6. Informative Mutational Signatures

For an explainable AI (XAI) algorithm to provide a valid explanation, the identified relevant features must genuinely contribute to the model’s prediction. If this is the case, removing these features should significantly reduce the model’s accuracy. This principle was tested by Samek et al. [48] and Bach et al. [45] using the “pixel flipping” method, which evaluates model performance after sequentially removing features deemed relevant. This approach enables the identification of the most effective algorithm for explaining the classifier’s decision-making process. Their study compared LRP with several other XAI methods and concluded that LRP not only outperformed the alternatives but also exhibited additional advantages. Specifically, they analyzed the complexity of the generated explanatory heatmaps and found that LRP-produced heatmaps had lower noise levels and identified a more concise set of relevant features. This enhances the interpretability of LRP-based explanations. These characteristics are crucial for the application of an XAI algorithm to a complex subject such as MS, where validation is more challenging compared to image-based tasks. In image analysis, the individual importance of features is intuitively understood, whereas in life science research, feature relevance is less straightforward and requires robust interpretability.

To generate our informative mutational signatures, we used the frequency information from the mutational signatures and extended them by the relevance calculation of the LRP. The informative mutational signatures are based on a combination of the mean mutational signature of the cancer-type with the positive median importance values of the features. The mutational signature of a cancer type is calculated by taking the mean values of the corresponding samples for all 96 trinucleotide contexts. The importance values are calculated for all features in every sample of this cancer type. Afterwards, the median value of all samples per feature is calculated. It includes, therefore, the information of which features are the most important for the classification of this cancer type. Other than regular mutational signatures, it is not solely focused on the frequency of the mutation, but also takes the information produced by the LRP into account.

To perform a comparison between informative mutational signatures, we utilize the normalized positive LRP relevance values for each cancer type to compute a weighted sum of absolute feature distances. This distance is then normalized by dividing by its maximum value, defined as the sum of the two highest LRP values for each cancer type. For comparison to standard mutational signatures from the COSMIC database (https://cancer.sanger.ac.uk/cosmic/ (accessed on 8 October 2024) [49]), the informative mutational signatures are used to calculate all versus all pairwise comparisons. This approach allowed us to identify the COSMIC signature combination that overlaps best with the frequency. The frequencies are further weighted by the LRP relevance of the informative mutational signatures. We define a combined COSMIC signature as fitting an informative mutational signature if its distance is lower than 5% of its maximal distance.

## 3. Results

### 3.1. Challenges to Discriminate Cancer Types Based on Standard Mutational Signatures

**Figure 1:**
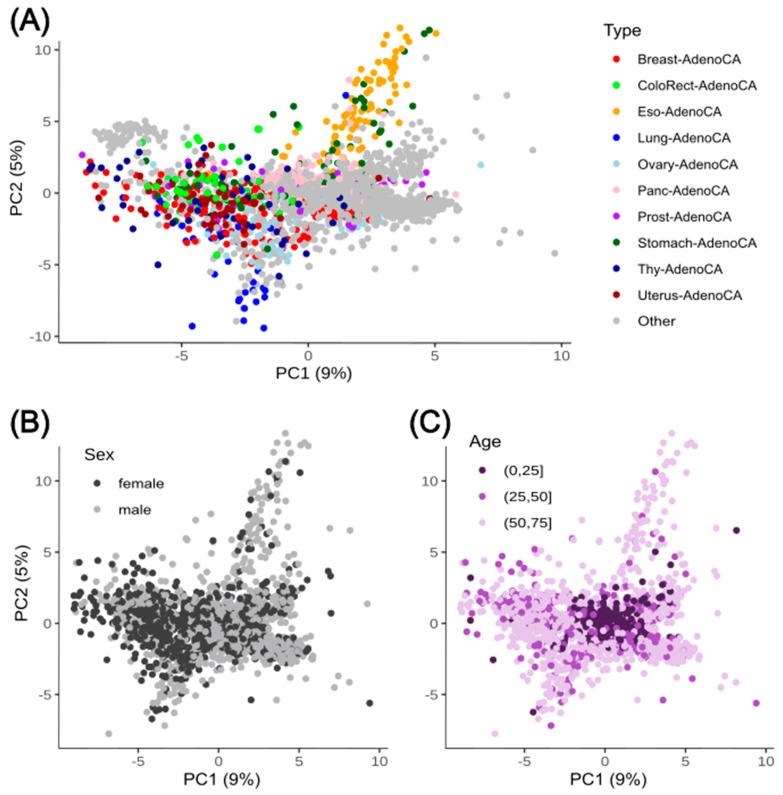
PCA of cancer samples based on their mutational signatures in the 3-nucleotide context. PCA of the 2592 samples of PCAWG based on their normalized mutational signature in 3-nucleotide context. (**A**) The samples are colored based on the different adenocarcinomas and grey is used for all other cancer types. (**B**) Samples are colored based on their biological sex (female: black; male: grey). (**C**) Samples are colored based on age groups of three intervals ((0,25] years in dark violet, (25,50] years in violet and (50,75] years in light violet).

Mutational signatures are frequency patterns of mutations and their nucleotide context that can be attributed to specific causes like DNA damage or DNA repair mechanisms. These causes vary significantly among different types of cancer but are also attributed partially to such causes. This variability makes it challenging to accurately identify and attribute specific signatures to particular cancer types, especially when multiple factors or processes contribute to the mutational landscape of a tumor. To identify the potential of mutational signatures to separate cancer types, we used the pre-processed PCAWG dataset (2592 patient samples of 24 different cancer-types) as input of unsupervised AI approaches to identify the main factors to separate these groups without giving labels. Therefore, we performed first a PCA on the z-score normalized frequencies of the mutational signatures (WGS 3-nucleotide context; Figure 1). In our analysis, we found two main principal components (PCs) explained less than 15% of the total variance (PC1: 9%; PC2: 5%) and could not be used to see clear clusters of cancer types. We found that mutational signatures alone were very difficult to explain separately by cancer type, except for some adenocarcinomas (Figure 1A). The esophagus and stomach adenocarcinomas seem to be clustered to some extent separately from the other cancer types. This correlation has also already been described for gastric and esophagus adenocarcinoma for specific driver gene mutations [50]. Nevertheless, the other adenocarcinomas from lungs were separated from them in PC2 and the prostate, pancreas, ovary, uterus and thyroid adenocarcinomas showed no separation within PC1 and PC2 from other cancer types. This gives us clues that patterns of mutations present in adenocarcinoma cells may help researchers to identify potential environmental exposures, DNA repair defects, or inherited genetic predispositions that contribute to the development of these tumors. Furthermore, we checked for potential confounders like sex (Figure 1B) or age (Figure 1C) within the first two PCs to exclude them as the main source for the 15% explained variability. When focusing on the sex of the patients, the samples showed no separation based on male or female. For the age grouping, a relatively tight cluster can be observed for the youngest age group (0 years to 25 years). The overlap between the four different age groups is still clearly visible. Based on this, it seems that biological mechanisms like DNA-repair mechanisms play a bigger role for the mutational signature, but the complexity and interplay of these mutations allow no clear, unsupervised separation.

Since PCA revealed neither clear separation nor explainability of the total variance within the PCAWG dataset, we investigated whether other unsupervised AI methods like hierarchical (Supplementary Figure S1) or k-means (Supplementary Figure S2) clustering can identify clearer trends in average mutational signature patterns across cancer types. Averaging led to the clustering of some primary sites like lung (AdenCA and SCC), lymph (CLL and BNHL) or parts of the CNS and adenocarcinomas. Most clusters showed no clear grouping of similar cancer types or primary sites. For the k-means clustering, nine clusters were identified but three of them contain a single cancer type. Clearly separated clusters showed a mix of different cancer types, especially for the adenocarcinomas. In our analysis, we found Lung-SCC and Lung-AdenoCA (same primary site) were clustered closely together as a single cluster. For others like CNS-Medullo and CNS-GBM (same primary site), or LymphCLL and LymphBNHL, we found in their clusters also adenocarcinomas and other cancer types assigned. In total, the unsupervised AI approach leads to no clear grouping based on meta-data (sex or age), cancer type or primary site, given the point mutation information in the 3-nucleotide context.

**Figure 2:**
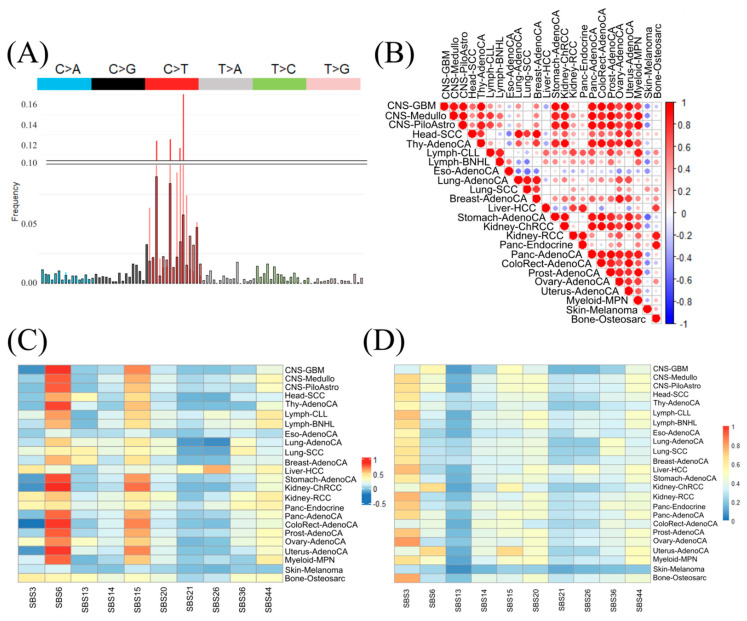
Correlation of mutational profiles in cancer types. (**A**) Example mutational profile signature of Thy-AdenoCA, overlapped with SBS6. The lower portion is scaled from 0 to 0.1, with both signatures overlayed. The Thy-AdenoCA is outlined in black. The upper portion is scaled from 0.10 to 0.175. (**B**) Dot heatmap of Pearson correlation between the different cancer types sorted by their primary site location within the human body. (**C**) Pearson correlation heatmap (−1: anticorrelation; 0: no correlation; 1: correlation) of the mutational profiles of cancer types to COSMIC signatures related to repair mechanisms (SBS3, SBS6, SBS13, SBS14, SBS15, SBS20, SBS21, SBS26, SBS36 and SBS44). (**D**) Heatmap of the summed-up feature-wise absolute correlation (0: no correlation; 1: complete correlation) of cancer types and the COSMIC signatures related to repair mechanisms.

To test confounders based on biological mechanisms or environmental effects (smoking, UV), we generated mutational signature profiles in the 3-nucleotide context for each cancer type and transformed it into a percentage-based profile (exemplified for LiverHCC in Figure 2A). The Pearson correlation of mutational signatures between cancer types showed a high variation within similar primary sites and cancer types (Figure 2B). Looking at correlations between cancer-type mutational signatures, a few groups become visible. On the one hand, the three CNS cancer types, for instance, showed high correlation with each other, but also with the seven of the eight adenocarcinomas (including Thy-AdenoCA, Stomach-AdenoCA, Panc-AdenoCA, ColoRect-AdenoCA, Prost-AdenoCA, Ovary-AdenoCA and Uterus-AdenoCA). On the other hand, Eso-AdenoCA shows low correlation with any of the cancers, same as Skin-Melanoma. Also, different cancer types of the same primary site as kidney (Kidney-ChRCC, Kidney-RCC) or pancreas (Panc-Endocrine and Panc-AdenoCA) show low to no correlation.

Next, we compared the mutational signatures of the cancer types to the known mutational signatures (single base substitutions; SBS) from the COSMIC database (86 known mutational signatures; https://cancer.sanger.ac.uk/signatures/sbs/ (accessed on 8 October 2024) [49]; Supplementary Table S1). When inspecting the overall maximum Pearson correlation between COSMIC and cancer-type mutational signatures, the values ranged from ~−0.008 to ~0.96. Thirteen of the 24 cancer types had their highest correlation with SBS1, a signature characterized by their C>T mutations. Signature SBS1 is clock-like and related to cell division and the mitotic clock and mentioned in respect to some cancers [51]. Other very high correlations were between Skin-Melanoma and SBS7a (0.96), a signature known to be associated with UV light exposure. When focusing on DNA repair mechanism signatures, two signatures (SBS6 and SBS15) seemed to be highly correlated to more than 12 cancer types (correlation above 0.5) using the overall mutation frequency pattern distance (Figure 2C). SBS6 is associated with DNA mismatch repairs [52] and characterized by C>T mutations, similarly to SBS1. There were no similar correlation values for SBS6 in relation to the same types of cancer (Eso-AdenoCA: 0.18 and Uterus-AdenoCA: 0.82) or the same tissue (Panc-Endocrine: 0.43; Panc-AdenoCA: 0.73). Interestingly, for SBS15, we observed a similar correlation pattern to the cancer types, which can be related to their similar C>T mutation pattern characterization like SBS6. As the overall Pearson correlation is not robust to outliers within the mutational signatures, we checked for the robustness using an absolute feature-wise correlation (similar to SigProfiler; [20]; Figure 2D). The DNA repair mechanisms SBS6 and SBS15 are less prominently correlated but instead SBS3 showed high correlation between our analyzed cancer types. SBS3 is associated with BRCA1 and two mutations and is related to small indels and genome rearrangements. In contrast, SBS26 (T>C mutations) shows still only a raised correlation with Liver-HCC and Skin Melanoma showed no correlation with the characterized COSMIC SBS for DNA repair.

In summary, we noticed that even within a single type of cancer, there can be significant heterogeneity in the mutational signatures among patients and they share similarities to multiple cancer types, reflecting putative common mechanisms of DNA damage and repair across different tissues. This complexity of various factors influencing mutational signatures could not be resolved by unsupervised AI models or reflected in correlation analysis. Our preliminary results show the challenges of distinguishing cancer types using mutational signatures as a discriminative factor.

### 3.2. Supervised ANNs Showed Best Learning Performance on WGS Mutational Signatures

As a next step, we wanted to add labels like cancer type and primary site to AI models allowing supervised training. This was already successful using a mix of topological mutation information and multiple mutational signature contexts [35]. To generate robust AI models, we performed a 10-fold cross validation for each grid-searched ANN architecture and used the optimal ANN for the final training and validation (Table 3). As input, we used for each approach the same 10-fold cross datasets (MS: 150 features of 1- to 3-nucleotide contexts; Bins: 2896 features from topological information). In contrast to the previous approach, we extracted the input features from the whole genome (WGS), the exome (WES) as well as the intronic and intergenic genome parts (WIIS) to see changes in the overall prediction accuracy (Table 3).

Comparison of the MCC and F1 scores of the 10-fold cross validation showed that the F1 score overall is lower than the MCC, which can be explained by the highly imbalanced numbers of patients for the 24 cancer types (Supplementary Table S2). Furthermore, the 10-fold cross-validation clearly shows that for each of the three approaches the prediction accuracy was in the same range (±5% MCC and F1, Table 3). The average MCC score for the WGS (0.685) was only slightly better in comparison to the WIIS (0.641) over all 10-fold cross-validations. In contrast, the difference in the average MCC from the WES (0.457) was higher than 0.2. In summary, the mutational information within the intronic and intergenic seems to be more important for discriminating cancer types in comparison to the exome information. Nevertheless, as the WGS approach had a higher accuracy compared to the WIIS, we can assume that the exome contains additional non-redundant information to improve the accuracy of the ANN model. This can also be seen in the prediction accuracy of the single cancer type (Supplementary Figure S3). Prediction of CNS-PiloAstro relied more on the intronic and intergenic mutation information, whereas esophagus adenocarcinoma had good prediction accuracy in all three models.

**Figure 3:**
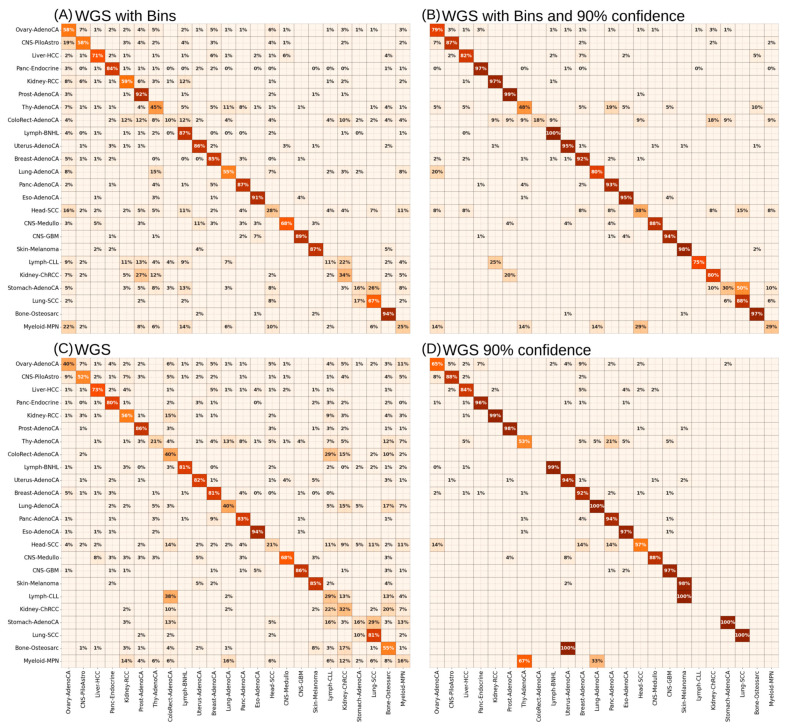
Summarized prediction accuracy of cancer types by the ANN. The aggregated confusion matrices of ANNs trained on (**A**,**C**) all or only the 90% confident (**B**,**D**) examples with WGS_MS + Bins (**A**,**B**) or only WGS_MS (**C**,**D**) features as input. The color coding from 0% (beige) to 100% (dark red) accentuates the prediction accuracy.

Besides the performance of cancer-type prediction using different genome parts, we also wanted to analyze the performance differences using all features (MS + Bins) or solely the mutational signature information (MS). Using solely mutational signature contexts (MS) led to a slight drop in the MCC scores of 0.033 (WGS) and 0.012 (WES). In contrast, the MCC score of the WIIS slightly increased (Supplementary Table S2). As the WGS input had the best overall accuracy for all predictions, we were interested in the single prediction accuracy for the cancer type and additionally performed the analysis a second time, with only a subset of predictions with a softmax output above 0.9 to train only on the high-confidence labels (Supplementary Figure S4). This led to four models differing in input features (MS + Bins and MS only) as well as using all labels for the training or only those with confidence above 90% (Figure 3).

The aggregated balanced accuracy of the confusion matrices of the ANN trained with WGS_MS + Bins (Figure 3A) on the validation sets ranged between 10% (ColoRect-AdenoCA) and 94% (Bone-Osteosarc). Comparing the accuracy differences between MS + Bins and MS only (Figure 3A,C) resulted in modest accuracy increases (less than 5%) for 14 out of 24 cancer types, indicating a low overall predictive relevance of Bin features. In general, 10 cancer types still had an accuracy above 80% to be correctly predicted. Exceptions were Ovary-AdenoCA (40% to 58%), Lung-AdenoCA (40% to 55%) and Bone-Osteosarc (55% to 94%) with an increased prediction accuracy using Bins. In contrast, the inclusion of the Bins also led to a large decrease in the prediction accuracy for Lung-SCC (81% to 64%) and ColoRect-AdenoCA (40% to 10%). In both cases, this was related to a higher percentage of specific misclassifications with other cancer types.

To generate a robust explainable AI model, it is important to have high accuracy in class prediction to be able to interpret the feature importance. For this reason, we performed a second iteration of the ANN training using only those examples with a >90% confidence (Figure 3B,D). For the WGS_MS + Bins and WGS_MS features, the prediction accuracy of 19 out of 24 cancer types could be improved. Notable accuracy improvements using only the secure training labels were observed for Ovary-AdenoCA (58% to 75%), CNS-PiloAstro (58% to 87%), Kidney-RCC (59% to 97%), Lung-AdenoCA (55% to 80%), Lymph-CLL (11% to 75%), and Kidney-ChRCC (34% to 80%). These improvements can largely be attributed to the significant reduction in uncertain predictions, with 50–90% of data points excluded in the high-confidence subset. As the addition of topological information (Bins) seems to have no major effect on prediction improvement, the combination of only secure training predictions (≥90%) led to two cancer types with missing samples (Kidney-ChRCC and ColoRect-AdenoCA) as well as three highly misclassified cancer types (Myeloid-MPN, Bone-Osteosarc and Lymph-CLL; Figure 3D). For the combination of topological information (Bins) and mutational signatures (MS), we exhibited for only five types of cancer (Thy-AdenoCA, ColoRect-AdenoCA, Head-SCC, Stomach-AdenoCA, and Myeloid-MPN) an accuracy level below 50%, with not so clear and drastic misclassifications. Given our focus on general cancer biomarkers and prediction accuracy, we concentrated our evaluation using XAI on the model trained with WGS_MS + Bins and a 90% confidence threshold to minimize biological variability.

### 3.3. XAI Models Allow Generation of Informative Mutational Signatures as a Source of Biologically Informed Diagnostics

**Figure 4:**
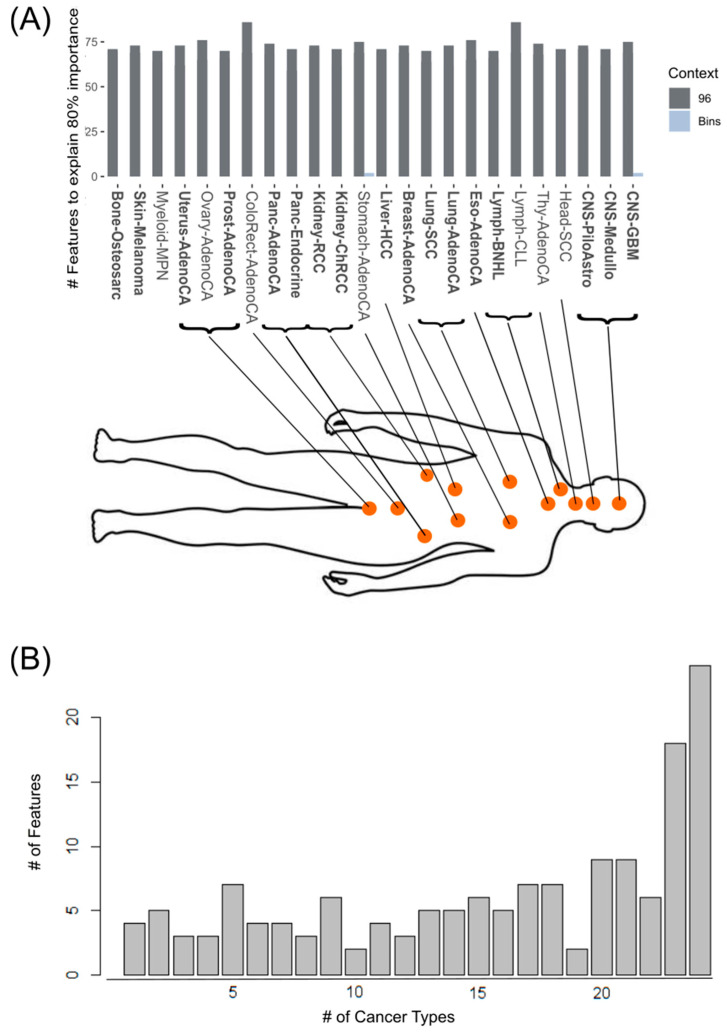
Most important features per cancer type. The features are sorted by their importance and (**A**) all features are summed up to 80% of the total importance (dark grey: 3-nucleotide context mutational signature feature; light grey: topological information Bin feature). The cancer types are sorted based on their primary site in the human body (bone, skin, myeloid are listed left as no clear assignment is possible). The cancer types with prediction accuracy > 80% are written in bold. (**B**) Number of common important features shared between cancer types.

As the single cancer-type prediction with the different input features suggested, the redundancy of information content between mutational signatures and topological information, we added the LRP as the explainable component to the ANN model. In addition to this overall explainability, the LRP allowed us to identify specific important features in the bins and the mutational signatures. This allowed us to extract for each single type of cancer the most influential input features for accurate prediction (Figure 4). The cancer types of Bone-OsteoSarc, Ovary-AdenoCA and Lung-AdenoCA were shown to be positively influenced by the prediction accuracy adding the topological information (Figure 3B,D). Nevertheless, none of these three cancer types had a specific topological information feature as one of the Top 50 important features (Supplementary Tables S3–S26). Only Stomach-AdenoCA and CNS-GBM had topological information features (Bins) in the Top 50 important features (Figure 4A).

The XAI model revealed that the most important features are mutation types from the 3-nucleotide and 2-nucleotide context (Supplementary Tables S3–S26). Overall, the single cancer types need between 59 and 87 important features to achieve a total of 80% importance (Figure 4A). Thereby, Head-SCC (59), Panc-Endocrine (59), CNS-Medullo (62), Lung-AdenoCA (62), Kidney-ChRCC (62) and Uterus-AdenoCA (62) seem to need less mutational signature features compared to ColoRect-AdenoCA (86) and Lymph-CLL (86). The majority of the 150 features were common in more than 20 cancer types (Figure 4B), of which 24 mutations in the 3-nucleotide context are considered as most important in all cancer types (Supplementary Table S27). Interestingly, only four (two Bin and two MS) of the 150 features are exclusive for one specific cancer type (Figure 4B). Two mutations that are specifically important for only one cancer-type prediction are T[T>A] mutations, for Eso-AdenoCA, and T[T>A]T mutations for Prost-AdenoCA. The other [T>A]T mutation can be used to predict Kidney-ChRCC and Liver-HCC. In contrast the T[C>T]A, T[C>T]T and [C>T]A mutations are all important for predicting Panc-Endocrine and Prost-AdenoCA. The high accuracy in correctly predicting Panc-Endocrine and Prost-AdenoCA is based more on the composition of important mutations in the mutational signatures like G[T>G] and [T>G] for Panc-Endocrine and [C>T], T[C>T] and T[C>T]C for Prost-AdenoCA (Supplementary Table S27). Furthermore, we observed that the different nucleotide contexts also had redundant information.

**Figure 5:**
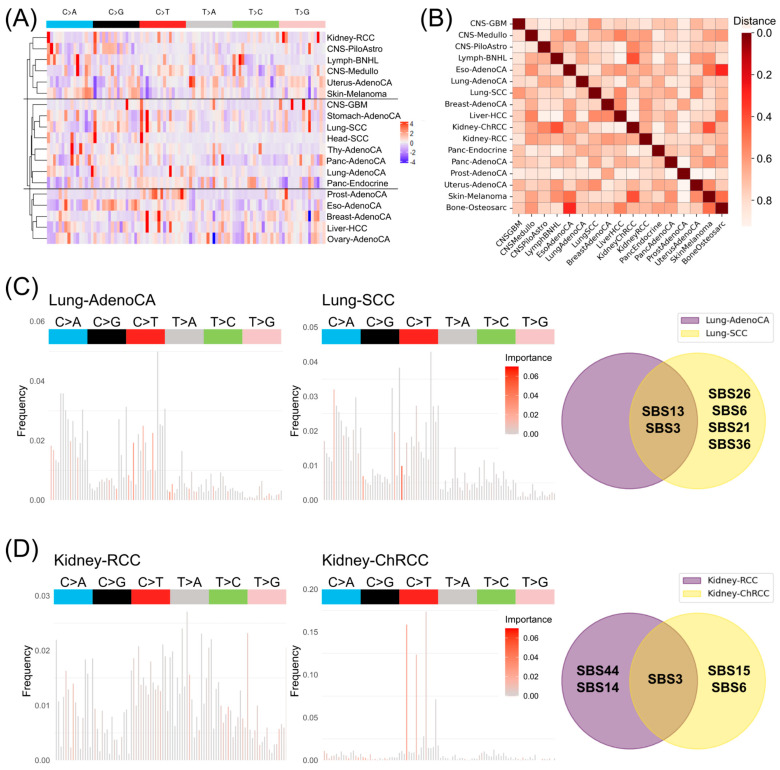
Informative mutational signatures for biologically informed diagnostics. (**A**) Hierarchical clustered cancer types (>80% accuracy) based on important feature similarities within the informative mutational signatures. (**B**) Heatmap of absolute differences in relevance scores between informative mutational signatures of cancer types. Informative mutational signatures of (**C**) Lung-AdenoCA and Lung-SCC as well as (**D**) Kidney-RCC and Kidney-ChRCC. The bars show the mean mutational profile, while the coloring highlights the top most important features. The Venn diagrams represent the common and specific DNA repair mechanism SBS from the COSMIC for the informative mutational signatures from (**C**,**D**) to separate the cancer types of the same primary sites.

In principle, the importance values of the single features of the informative mutational signatures (Figure 5A) as well as the relevance score distance (Figure 5B, Supplementary Table S28) showed no clear grouping of cancer types or primary sites. Certain cancer types of similar origin, like Lung-AdenoCA and Lung-SCC or Panc-AdenoCA and Panc-Endocrine were assigned to the same cluster but there are also cancer types like CNS-GBM separated from the other two CNS cancer types. The adenocarcinomas meanwhile are scattered across the three clusters (cluster1: Uterus-AdenoCA; cluster2: Stomach-AdenoCA, Thy-AdenoCA, Panc-AdenoCA, Lung-AdenoCA; cluster3: Prost-AdenoCA, Eso-AdenoCA, Breast-AdenoCA, Ovary-AdenoCA). This could be also seen in the statistical analysis of the top 5 single important mutations per cancer type versus all other cancers (Supplementary Table S29). Here, most of the single mutations were significant to separate the specific cancer from the other cancer types but the pairwise cancer-type testing revealed that many specific mutations cannot be exclusively used for cancer-type prediction. The additional relevance of the informative mutational signatures in contrast to the standard mutational signatures can be seen in the direct comparison of cancer types of the same primary site (Figure 5C,D). While the standard mutational signatures are comparing solely the frequencies of the single mutations allow the informative mutational signatures to weigh the most relevant features for the correct cancer-type prediction. In the case of similar mutational signatures, the additional information layer allows a more precise and specific assignment of existing SBS from the COSMIC database. In the example of lung cancer types (Lung-AdenoCA and Lung-SCC), the mutational signatures have similar frequencies, but the relevant feature-weighing approach allows us to assign specific impaired DNA repair mechanisms to Lung-SCC (Figure 5C). Four of the six assigned DNA repair mechanism SBSs of the COSMIC were specifically assigned to Lung-SCC. In the case of more divergent mutational signatures like Kidney-RCC and Kidney-ChRCC, the assignment of specific impaired DNA repair mechanisms allows even more clear biologically informed diagnostics (Figure 5D).

### 3.4. Informative Mutational Signatures Contain Non-Redundant Information in Comparison to Cancer Driver Gene Mutations

**Figure 6:**
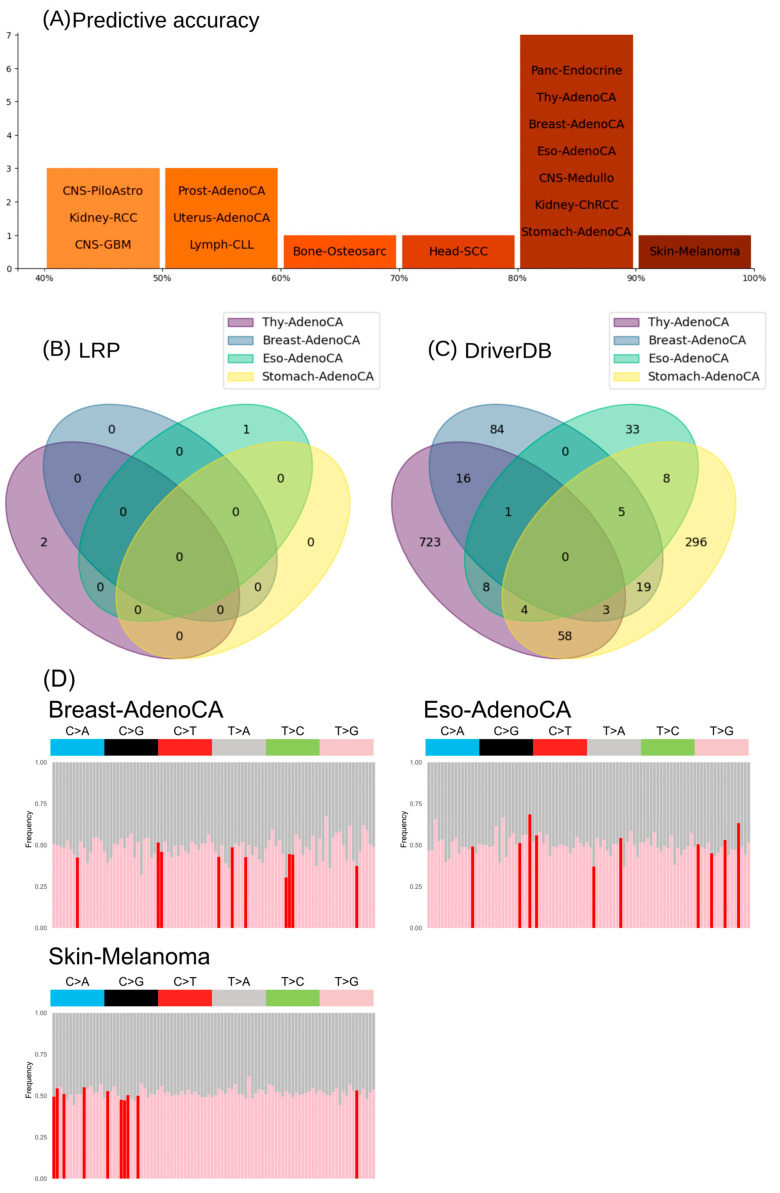
Gene mutation signatures to predict cancer types. (**A**) The prediction accuracy of the ANN for each cancer type. Venn diagrams comparing the important genes of the LRP-ANN (**B**) to the known driver genes of the DriverDB (**C**) for thyroid, breast, esophagus and stomach adenocarcinoma. (**D**) Visualization of the frequency of important features of the informative mutational signature in the known driver genes of the DriverDB (red: top 10 most important mutational signature feature). The proportion of each of the 3-nucleotide context mutational signatures is plotted between the driver genes (pink, red) and the remaining parts of the genome (grey).

In current clinical approaches for cancer diagnostics, important driver genes and their mutations are predominantly used; we wanted to see if our informative mutational signatures are more an alternative to specific gene mutations or add new information to improve cancer prediction (Figure 6). Therefore, we calculated for each gene the number of mutations (MS_GeneM) and used the same ANN and cross-validation setup to predict the 24 cancer types and used the LRP to extract the importance of the single genes for prediction accuracy (Supplementary Table S30). Overall, the prediction accuracy was above 80% for eight cancer types (including four adenocarcinomas; Figure 6A and Supplementary Figures S5 and S6). To identify new potential driver genes with the LRP-ANN, we defined cutoffs based on this threshold, which is based on the frequency of driver genes reported by Michael S. Lawrence et al. [47]. If the gene and its mutations were in more than 20% (respectively, 10% or 2%; Supplementary Figure S5) of the patients’ samples, found as one important feature based on our quantitative-LRP score to correctly assign the cancer type, we called it a potential cancer driver gene. Within the eight highly accurately predicted (>80% accuracy) cancer types, a maximum of two genes were identified by the quantitative-LRP score to be important (Supplementary Table S31). In Thy-AdenoCA, the genes NRXN3 and LRP1B exceeded this threshold, as well as PCDH15 in Eso-AdenoCA and Skin-Melanoma. A comparison of the quantitative-LRP-scored genes to the known driver genes (DriverDB, [44]) for the four adenocarcinoma showed much lower numbers of potential driver genes (Figure 6B,C). Furthermore, only our quantitative-LRP-scored potential driver gene LRP1B was overlapping the known driver genes of the analyzed cancer types (Supplementary Figure S7).

Based on these results, it seems that the prediction of cancer types solely on the mutation information of the gene regions gives less accurate information than using the WGS mutational signatures. Further, the important genes for the cancer-type classification of the LRP-ANN are barely overlapping with the known driver genes and the low number of important genes and the low quantitative-LRP scores suggest limited confidence in this XAI model with gene mutations as features. To support this theory and prove that our informative mutational signatures add additional information for the prediction of cancer types, besides driver genes, we analyzed the mutation distribution between WGS and driver genes (Figure 6D, Supplementary Figure S8). We focused on the three cancer types (Breast-AdenoCA, Eso-AdenoCA and Skin-Melanoma) that had a high predictive accuracy (>80%) with the gene information (Figure 6) and WGS (Figure 3) as well as at least one known driver gene. In general, most mutations in the informative mutational signatures seemed to occur equally frequently in the driver genes and the remaining WGS regions. Furthermore, the most important features of the informative mutational signatures showed no obvious overrepresentation in the known driver genes (Figure 6D, red). Based on these observations, it seems that the informative mutational signatures reveal a new mechanistic layer in cancer development. In contrast to driver genes pinpointing single-point mutations, the informative mutational signatures allow a more cell-wide perspective on the impairment of DNA repair mechanisms for the cancer-type prediction.

## 4. Discussion

Accurate cancer-type prediction, especially in the context of typing, grading or staging [53] is of major importance in the clinical environment, as early diagnosis would allow better healing chances and provide longer life [54,55,56]. Furthermore, cancer therapy and curation are very cost intensive and unsuccessful therapies lead to time loss for the patients and increased costs [57,58]. As many pathologists rely on morphology, it could be shown that such techniques, even by experts, result in accuracy between 49% and 76% [59]. The usage of tissue-specific antigens via IHC can increase the prediction accuracy to 83% but this is known to be confounded by the loss of antigens in poorly differentiated tumors [60,61,62]. Comparison of these accuracy values with our informative mutational signatures from the LRP-ANN model (Supplementary Figures S9 and S10) revealed that we could already exceed these accuracies for many of the cancer types (Figure 3).

As alternatives to IHC and phenotypic diagnosis, molecular profiling of tumors using mRNA or miRNA expression can be used [2]. In these cases, miRNA and mRNA expression signatures can be used to predict primary sites and cancer types correctly in 76% to 89% of cases [63] and can even predict poorly differentiated tumors [64]. As these approaches are analyzed using standard bioinformatics and statistical analysis, most of them focus on a few pairwise cancer-type comparisons. Further, these RNA or protein expression changes can be affected by different environmental factors and because of this, they show a higher variability and heterogeneity [65]. In contrast to these approaches, the definition of informative mutational signatures tackles the source of cancer disease and, because of this, seem to be more robust. Even with a 10-fold cross-validation of the PCAWG for 24 cancer types, from 2592 patients, we observed a maximum discrepancy of 5% overall in the prediction (Table 2). As WGS and the extraction of mutational signatures recently became more affordable, the route goes in the direction of routine WES or WGS analysis for cancer prediction in the UK [66]. WES approaches in combination with machine learning models have already achieved good results for different numbers of primary sites in their overall prediction accuracy of between 62% and 78% [32,67,68]. If studies about WGS primary sites were compared [35], it would be stated that the WGS data contain more useful information for the discrimination of cancer types and primary sites. In our study, we confirmed this hypothesis by using the same dataset and ANN model for the different parts of the genome (exome, genome, intron and intergenic parts; Table 2). Based on this, we found out that much information about the mutational signatures for predicting cancer types are in the intergenic and intronic regions (Supplementary Figure S3). Furthermore, we confirmed using LRP-ANN that the mutational signatures contain the most important information for accurately predicting cancer type, and that topological information (Bins), as in Jiao et al. [35], seems to be negligible for many cancer types, as driving factors (Figure 4). Nevertheless, multi-information datasets, such as TumorTracer [33] or Jiao et al. [35] as well as multi-omics [69], can be used to achieve high accuracy in cancer-type prediction. Our LRP-ANN model and the informative mutational signatures give first insights into non-redundant information gain using more than the 3-nucleotide context, while even the informative mutational signatures allowed the differentiation of cancer types at the same primary site (Figure 5) and between the same cancer types (Supplementary Table S29).

Currently, functional mutations across driver genes are used to predict cancer types but it could be shown already that these are not the best hallmarks for cancer-type prediction [30]. This is in line with our observations of using the mutations within genes alone to predict the cancer type, ending up with only eight accurate predictable cancer types without having clear important driver genes (Figure 6). In addition, driver genes are also not conserved within all databases [70,71] and specific panels exist to predict specific cancer types but do not exclude all other cancers. Also, the frequency of informative mutations show no clear relation to the known driver genes and driver mutations (Figure 6D). Not all the important mutations were over-represented in driver genes, nor were all mutations that were overrepresented in driver genes among the most important for the classification. Our informative mutational signatures allow us to assign specific impaired DNA repair mechanisms based on their mutation frequencies, weighing them according to their relevance (Figure 5).

Besides the somatic mutations for predicting cancer types and primary sites, there are also approaches known to diagnose cancers of unknown primary sites (CUPS) using methylation patterns or specific marker proteins. While IHC remains the clinical standard for the diagnosis of CUPS in clinical practice [72], it relies on specific marker proteins and limits its utility for biomarker discovery. Moreover, it is surpassed in predictive accuracy based on gene expression approaches for known primary sites [62] reported in a multi-center study that compared IHC and pathwork tissue of origin tests based on NGS. Moran et al. [73] demonstrated that epigenetic profiling of methylation patterns can be used to predict the tissue of origin of cancer (CUPS) with an accuracy of 87%. They utilized microarray-based DNA methylation data in conjunction with an RF classifier, leveraging the feature-importance scores to generate cancer-type-specific methylation profiles. While the analysis of somatic mutations, as used in our approach, do not enable the identification of CUPS, it does offer the distinct advantage of directly interrogating oncogenic mechanisms, in contrast to the indirect functional insights provided by methylation or gene expression data [72]. Both data sources, in combination with AI tools, allow the accurate detection of cancer types but differ in their potential for special kinds of cancers, like CUPS. Our informative mutational signatures (Figure 6) are not suitable for predicting CUPs but therefore add biologically relevant information about the dysfunctions in the DNA repair mechanisms and thereby may directly link the cancer-type prediction to potential therapeutic solutions in future. A multi-omics strategy that integrates these complementary molecular datasets in combination with XAI approaches could in future lead to the more accurate prediction of CUPS and cancer type, including biological information for more insights into the functional mechanisms.

As ML and DL approaches have been implemented to predict cancer types or primary sites [74,75,76] (Table 1), the improvement in our approach is the extraction of the most relevant somatic mutations per cancer type to add this information for the mutational signatures (Figure 4 and Figure 5). A comparison of the overall accuracy of the models reveals that our model exhibits a maximum discrepancy of 20% in comparison to other models. However, this discrepancy must be contextualized, as the latter is predicated on a mere 13 cancer types, whereas our model encompasses nearly twice the number of cancer types. DL models have been demonstrated to enhance the precision of predictions but lack a clear interpretation [35]. The problems with DL models lie in their black box behavior only giving confident outputs that may rely on Clever Hans predictors [43]. To overcome such problems with sensitive topics like medical applications, it is essential to build robust and explainable components to make the scores and additional values interpretable [77,78,79]. Our LRP-ANN revealed that topological information is only relevant for the cancer types Stomach-AdenoCA and CNS-GBM (Figure 4). In the other cases, our informative mutational signature was identified as the primary influencing factor on cancer-type prediction (Supplementary Tables S3–S26). Furthermore, the added information showed promising results for more biologically informed diagnostics in future, with the cancer-type prediction relying on the combination of mutation relevance and mutation frequency in the informative mutational signatures.

## 5. Conclusions

Overall, mutational signatures have great potential to increase the accuracy of cancer-type prediction, especially in combination with other information and methodologies. In our approach, we observed that the mutational signatures using the 1- to 3-nucleotide contexts alone had, apart from a few exceptions, more information content than topological information. Furthermore, we could show that it is essential to perform robust cross-validation and dataset processing as the imbalance between the PCAWG dataset and cancer-type rarity, in general, lead to Clever Hans predictions. Also, it could be observed that the intronic and intergenic regions seem to be crucial for the accurate prediction of cancer types. In addition, we could achieve a clear assignment of specific COSMIC SBS to cancer types, irrespective of their mutation frequency similarities, by adding the relevance of mutations to the mutational signatures. Through this, our newly presented informative mutational signatures could be used in future for more biologically informed diagnostics of cancer types relating to specific impaired DNA repair mechanisms. As mutational signatures are often related to malfunctions in DNA repair mechanisms, it seems not to be a clear one-to-one connection of cancer type and DNA repair mechanism but more like an interplay of different malfunctions and regulatory mechanisms. For the first time, our approach allowed us to not only train a DL model to predict cancer on WGS but also to set it in the context of a DNA repair mechanism SBS. Nevertheless, it seems that cancer types and primary sites have specific informative mutational signatures, which now can be analyzed, in order to integrate them in combination with driver genes for more accurate typing, grading and staging in future.

## Figures and Tables

**Table 1:** Overview of AI methods for cancer prediction based on NGS. Given are the first author, year, doi, AI method used and input data source.

Author	Year	Title	AI Method	Input Data Source
Khan et al.	2001	10.1038/89044 [27]	ANN/DNN	Expression (RNA-Seq)
Peng et al.	2006	10.1016/j.compbiomed.2005.04.001 [36]	SVM	Expression (Microarray)
Chen et al.	2015	10.1155/2015/491502 [32]	SVM	Somatic alterations
Yuan et al.	2016	10.1186/s12859-016-1334-9 [34]	ANN/DNN	Somatic alterations
Liu et al.	2019	10.3390/genes10100778 [37]	ANN/DNN	Expression (Methyl-Seq)
Jiao et al.	2020	10.1038/s41467-019-13825-8 [35]	ANN/DNN, RF	Somatic alterations
Mostavi et al.	2020	10.1186/s12920-020-0677-2 [28]	CNN	Expression (RNA-Seq)
Kim et al.	2020	10.1093/bioinformatics/btz772 [38]	SVM, RF, ANN/DNN	Expression (Single cell RNA-Seq)
Zelli et al.	2023	10.1186/s12967-023-04720-4 [39]	XGBoost	Somatic alterations
Darmofal et al.	2024	10.1158/2159-8290.CD-23-0996 [40]	RF, ANN/DNN	Somatic alterations
Alanazi et al.	2024	10.1016/j.sjbs.2023.103918 [29]	SVM, RF, ANN/DNN etc.	Expression (RNA-Seq)

**Table 2:** Input datasets. Naming conventions for the different input datasets and what data is included.

Dataset Name	Input
WGS_MS	Mutation counts in 3-nucleotide-, 2-nucleotide and 1-nucleotide context (mutation context) on all mutations across the genome
WGS_MS + Bins	Mutation context on all mutations across the genome + number of mutations in 1 Mbp-bins
WES_MS	Mutation context on all mutations in exonic regions
WES_MS + Bins	Mutation context on all mutations in exonic regions + number of mutations in 1 Mbp-bins
WIIS_MS	Mutation context on all mutations in intronic and intergenic regions
WIIS_MS + Bins	Mutation context on all mutations in intronic and intergenic regions + number of mutations in 1 Mbp-bins
WGS_GeneM	Mutation counts in 3-nucleotide-context on all mutations separated by genes

**Table 3:** ANN-model accuracy. Metrics collected for the validation. The values were calculated by taking the mean of the metric values for all output classes.

Dataset	Crossfold Iteration	Precision	Recall	F1 Score	MCC Score
WGS_MS + Bins	1	0.63	0.65	0.62	0.72
	2	0.61	0.61	0.60	0.67
	3	0.64	0.63	0.63	0.72
	4	0.61	0.61	0.59	0.67
	5	0.67	0.63	0.61	0.68
	6	0.68	0.64	0.65	0.72
	7	0.60	0.58	0.58	0.68
	8	0.57	0.59	0.56	0.66
	9	0.60	0.59	0.57	0.66
	10	0.61	0.61	0.58	0.67
WES_MS + Bins	1	0.47	0.43	0.44	0.48
	2	0.40	0.41	0.40	0.44
	3	0.40	0.41	0.39	0.43
	4	0.48	0.45	0.45	0.50
	5	0.49	0.43	0.44	0.46
	6	0.40	0.39	0.39	0.46
	7	0.52	0.42	0.44	0.46
	8	0.44	0.43	0.42	0.44
	9	0.47	0.45	0.44	0.47
	10	0.42	0.39	0.39	0.43
WIIS_MS + Bins	1	0.58	0.54	0.54	0.64
	2	0.54	0.54	0.52	0.61
	3	0.56	0.56	0.54	0.64
	4	0.56	0.54	0.53	0.63
	5	0.55	0.54	0.53	0.65
	6	0.53	0.54	0.52	0.63
	7	0.54	0.57	0.54	0.65
	8	0.55	0.55	0.53	0.64
	9	0.57	0.56	0.54	0.66
	10	0.60	0.55	0.55	0.66

## Data Availability

All datasets used in this publication were available from the PCAWG (https://dcc.icgc.org/, accessed on 7 October 2021). All codes of models and processed datasets can be accessed by the corresponding author and on github (https://github.com/Wombu/informative_mutational_signature, accessed on 26 February 2025).

## Supplementary Materials

The following supporting information can be downloaded at: https://www.mdpi.com/article/10.3390/cancers17111731/s1, Figure S1: The dendrogram displays the clustering of the samples using their trinucleotide mutation counts after z-score normalization; Figure S2: This graphic shows the k-means clustering of the z-score normalized mutation counts in trinucleotide context; Figure S3: The heatmaps shows the accuracy values for the best crossfold iteration using the ANN on three different datasets; Figure S4: Bar charts illustrating the confidence levels of predictions made by the ANN for models trained with WGS_MS + Bins (A) and WGS_MS only (B); Figure S5: This confusion matrix displays the balanced accuracy values of an ANN only trained with WGS_GeneM for all 24 cancer types; Figure S6: Bar charts illustrating the confidence levels of predictions made by the ANNs trained with WGS_GeneM; Figure S7: These venn diagrams show the overlap between the quantitative-LRP driver genes with an occurrence threshold of 20% based on Michael S. Lawrence et al. and the DriverDB gene list; Figure S8: The plots show the proportion of specific mutations in marker genes compared to the number of mutations in nonmarker genes for the cancer types that reached 80% accuracy using genes but were below 80% accuracy when using the ANN on WGS_MS + Bins; Figure S9: Informative mutational signatures, the bars show the mean mutational profile, while the coloring highlights the positive relevance scores of the mutation types; Figure S10: Informative mutational signatures, the bars show the mean mutational profile, while the coloring highlights the positive relevance scores of the mutation types; Table S1: Correlation of cancer type signatures with COSMIC signatures. Pearson correlation between the mean mutational signatures of each cancer type with the 86 known signatures in the COSMIC database; Table S2: Metrics for the unbinned training. Collected metrics for the validation while training on datasets using only mutation context. The values were calculated by taking the mean of the metric values for all output classes; Table S3: LRP relevance scores for Bone-Osteosarc; Table S4: LRP relevance scores for Breast-AdenoCA; Table S5: LRP relevance scores for CNS-GBM; Table S6: LRP relevance scores for CNS-Medullo; Table S7: LRP relevance scores for CNS-PiloAstro; Table S8: LRP relevance scores for ColoRect-AdenoCA; Table S9: LRP relevance scores for Eso-AdenoCA; Table S10: LRP relevance scores for Head-SCC; Table S11: LRP relevance scores for Kidney-ChRCC; Table S12: LRP relevance scores for Kidney-RCC; Table S13: LRP relevance scores for Liver-HCC; Table S14: LRP relevance scores for Lung-AdenoCA; Table S15: LRP relevance scores for Lung-SCC; Table S16: LRP relevance scores for Lymph-BNHL; Table S17: LRP relevance scores for Lymph-CLL; Table S18: LRP relevance scores for Myeloid-MPN; Table S19: LRP relevance scores for Ovary-AdenoCA; Table S20: LRP relevance scores for Panc-AdenoCA; Table S21: LRP relevance scores for Panc-Endocrine; Table S22: LRP relevance scores for Prost-AdenoCA; Table S23: LRP relevance scores for Skin-Melanoma; Table S24: LRP relevance scores for Stomach-AdenoCA; Table S25: LRP relevance scores for Thy-AdenoCA; Table S26: LRP relevance scores for Uterus-AdenoCA; Table S27: Mutational signature features shared as important features within the cancer-types. The cancers which consider it among the 80% most important are indicated with a 1; Table S28: Absolute distance between the relevance values of two informative mutational signatures; Table S29: Significance of single mutations. P-values for the Wilcox-tests on comparison of the mean number of the 5 most important mutation types for each cancer type with the other cancer types grouped together and with each cancer type separately. The *p*-values for the pairwise comparisons were adjusted using the Holm-Bonferroni method; Table S30: Quantitative LRP-Analysis for the ANNs trained with the MS_MGene dataframe; Table S31: Genelist of high predictive accuracy labels (>80%) from the Quantitative LRP analysis for ANNs trained with the MS_MGene dataframe.
