# Supplementary material for: Explainable AI Model Reveals Informative Mutational Signatures for Cancer-Type Classification
Source: Cancers (Basel). 2025 May 22;17(11):1731. doi: 10.3390/cancers17111731 (PMC12153866; doi:10.3390/cancers17111731)
Supplement: Supplementary file 1 [file cancers-17-01731-s001.zip › Supplemental Figures.pdf]

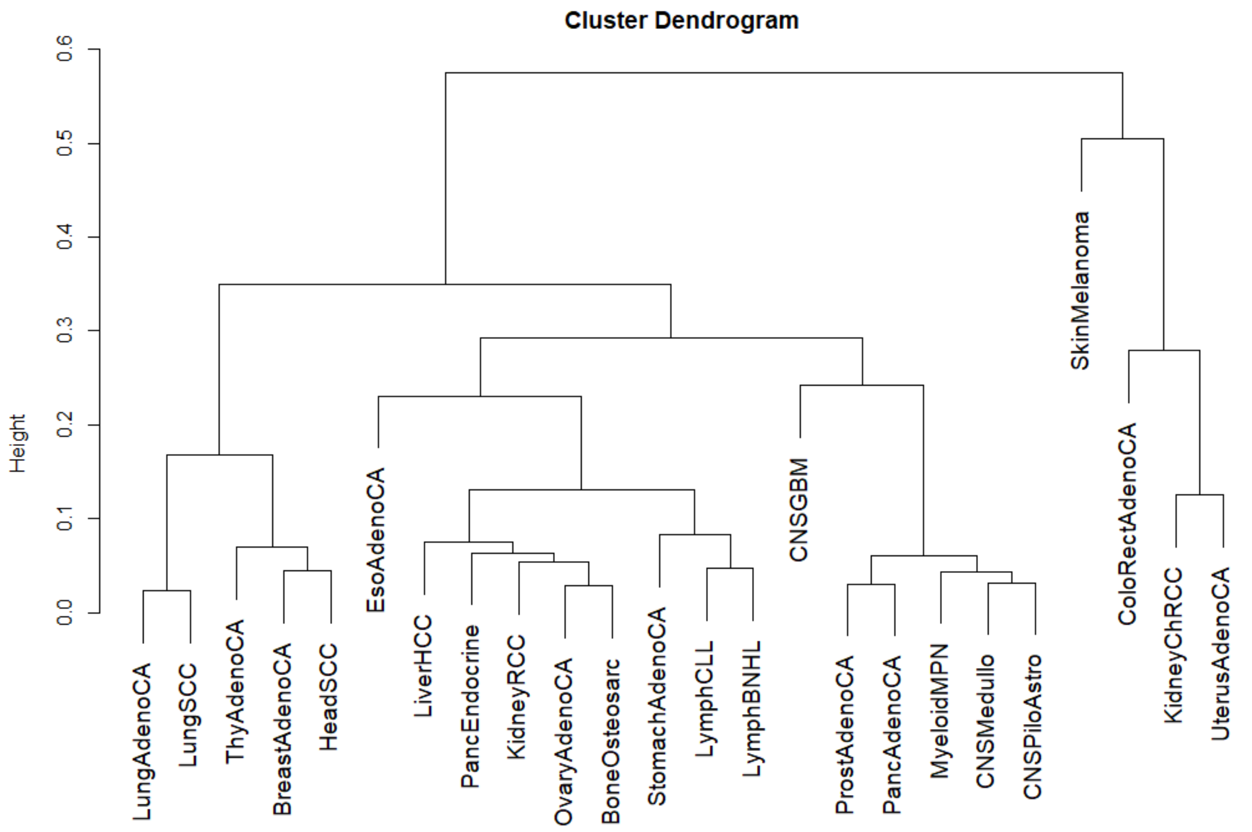

Supplemental Figure S1. The dendrogram displays the clustering of the samples using their trinucleotide mutation counts after z-score normalization.

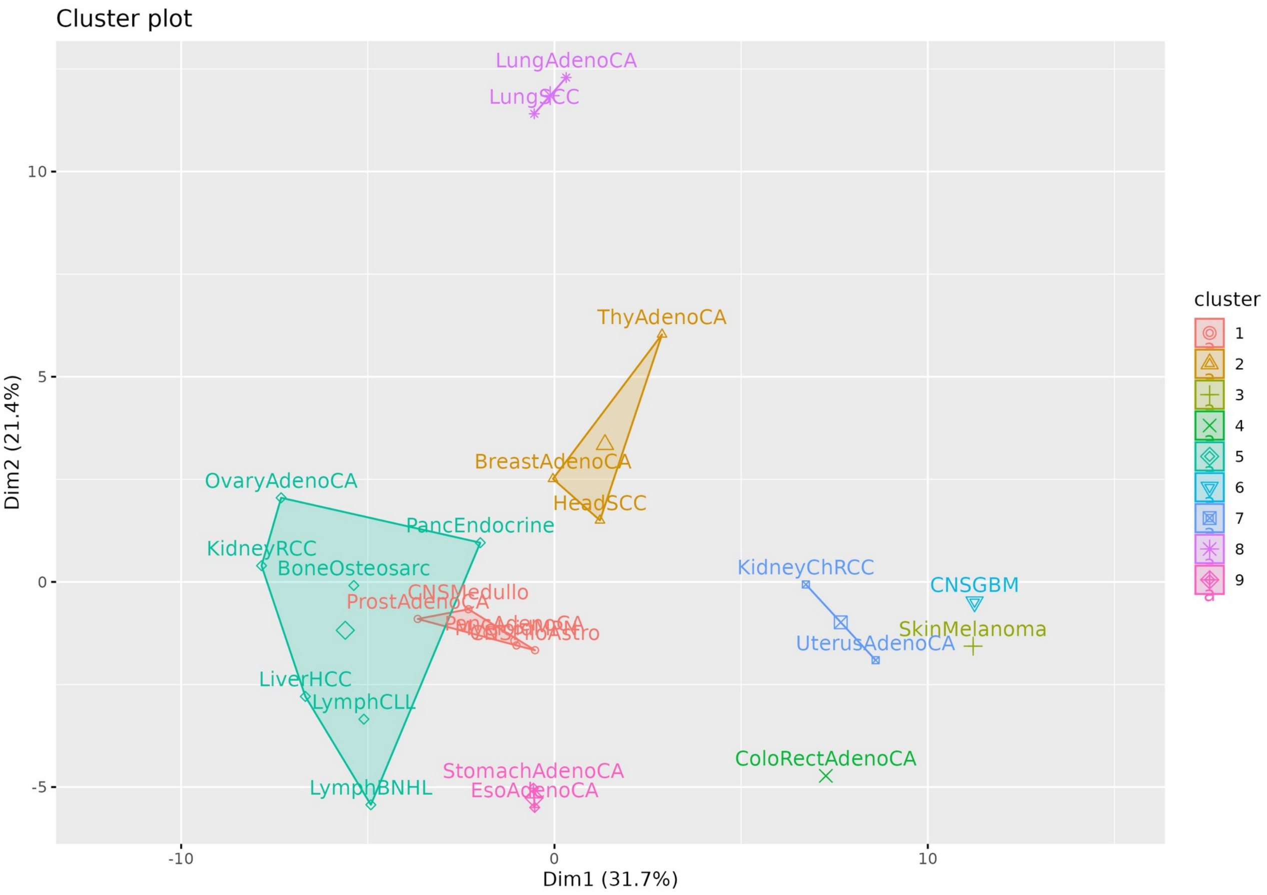

Supplemental Figure S2. This graphic shows the k-means clustering of the z-score normalized mutation counts in trinucleotide context.

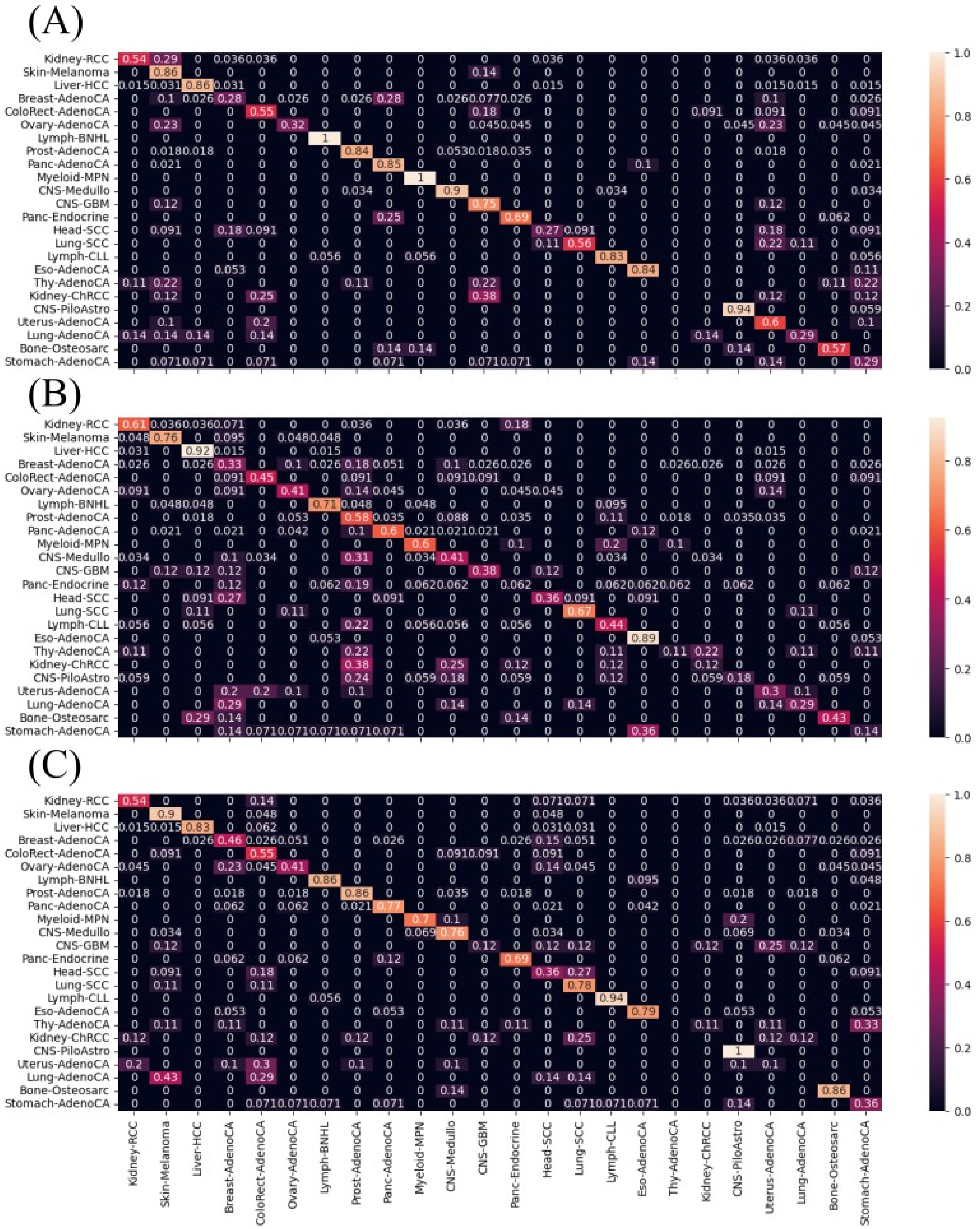

Supplemental Figure S3. The heatmaps shows the accuracy values for the best crossfold iteration using the ANN on three different datasets. (A) shows the training on the WGS_MS+Bins dataset, (B) the WES_ MS+Bins dataset and (C) the WIIS_ MS+Bins dataset.

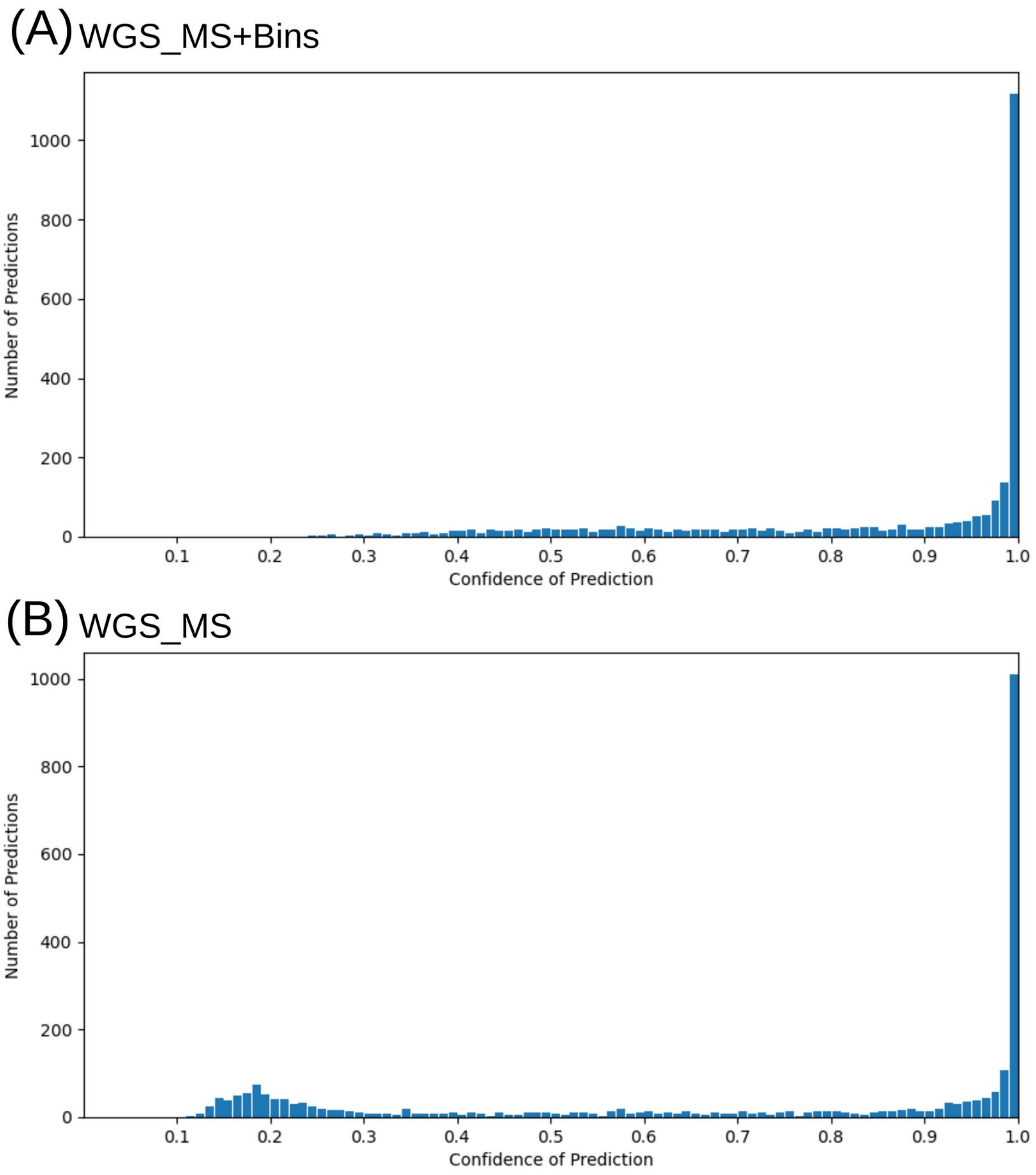

Supplemental Figure S4. Bar charts illustrating the confidence levels of predictions made by the ANN for models trained with WGS_MS+Bins (A) and WGS_MS only (B). Both charts demonstrate that the majority of predictions have a confidence level exceeding 0.9. In chart B, however, a small cluster of predictions shows lower confidence, ranging between 0.1 and 0.3.

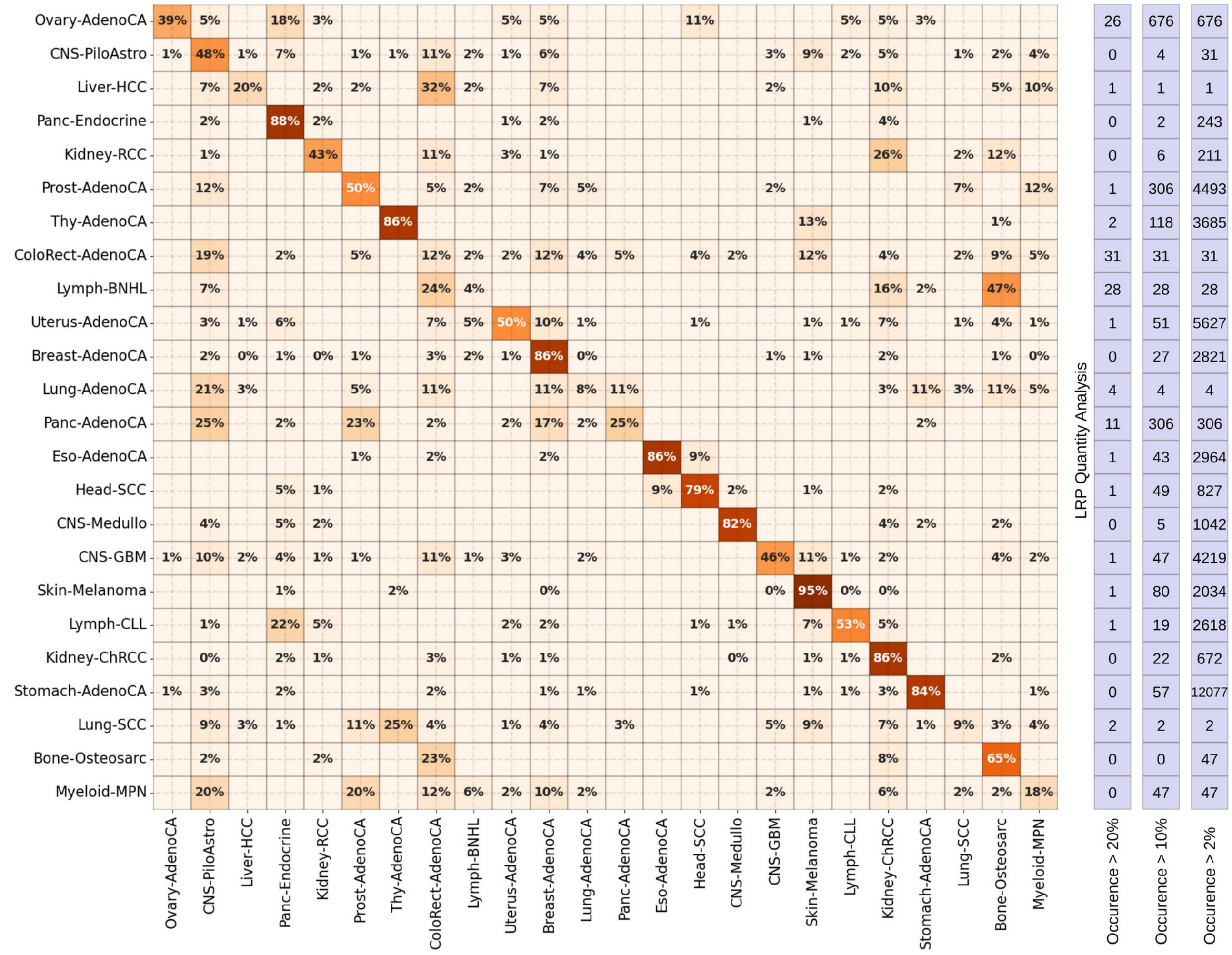

Supplemental Figure S5. This confusion matrix displays the balanced accuracy values of an ANN only trained with WGS_GeneM for all 24 cancer types. The quantitative-LRP analysis shows the occurrence of features for different thresholds based on the publication of Michael S. Lawrence et al..

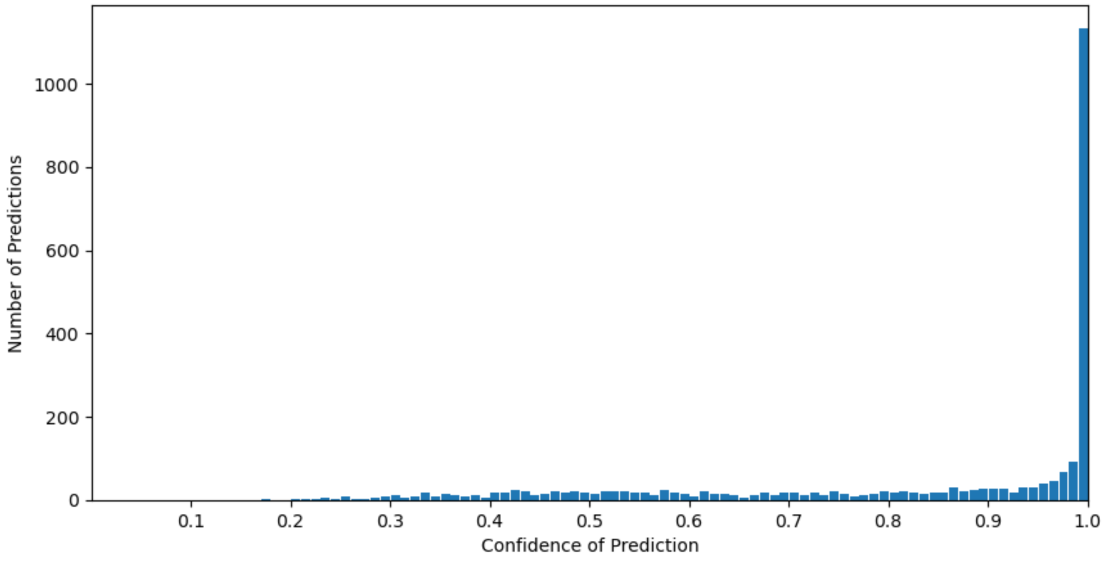

Supplemental Figure S6. Bar charts illustrating the confidence levels of predictions made by the ANNs trained with WGS_GeneM.

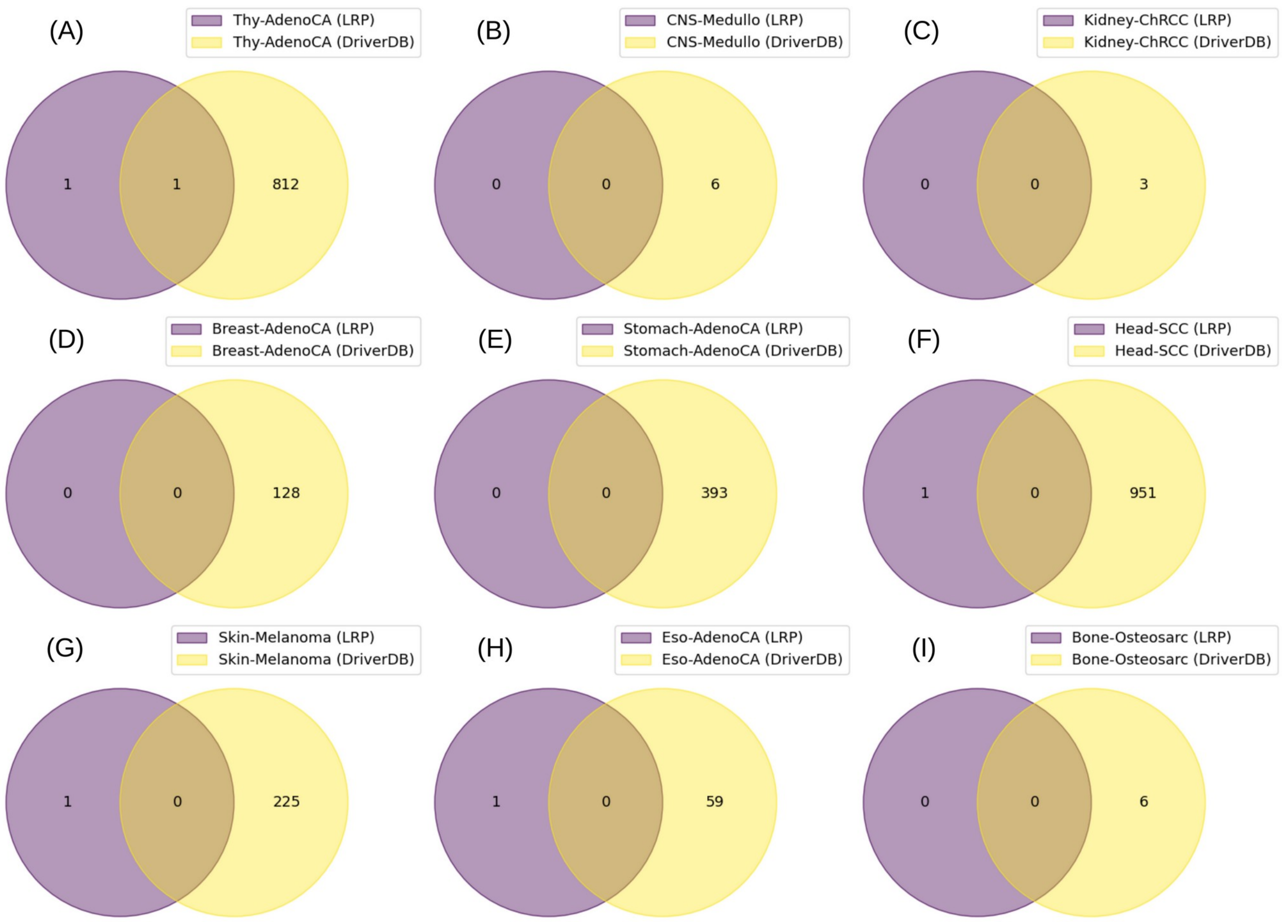

Supplemental Figure S7. These venn diagrams show the overlap between the quantitative-LRP driver genes with an occurrence threshold of 20% based on Michael S. Lawrence et al. and the DriverDB gene list. This grafik only incompases cancer types with an balanced accuracy above 60% from the ANN trained with WGS_GeneM.

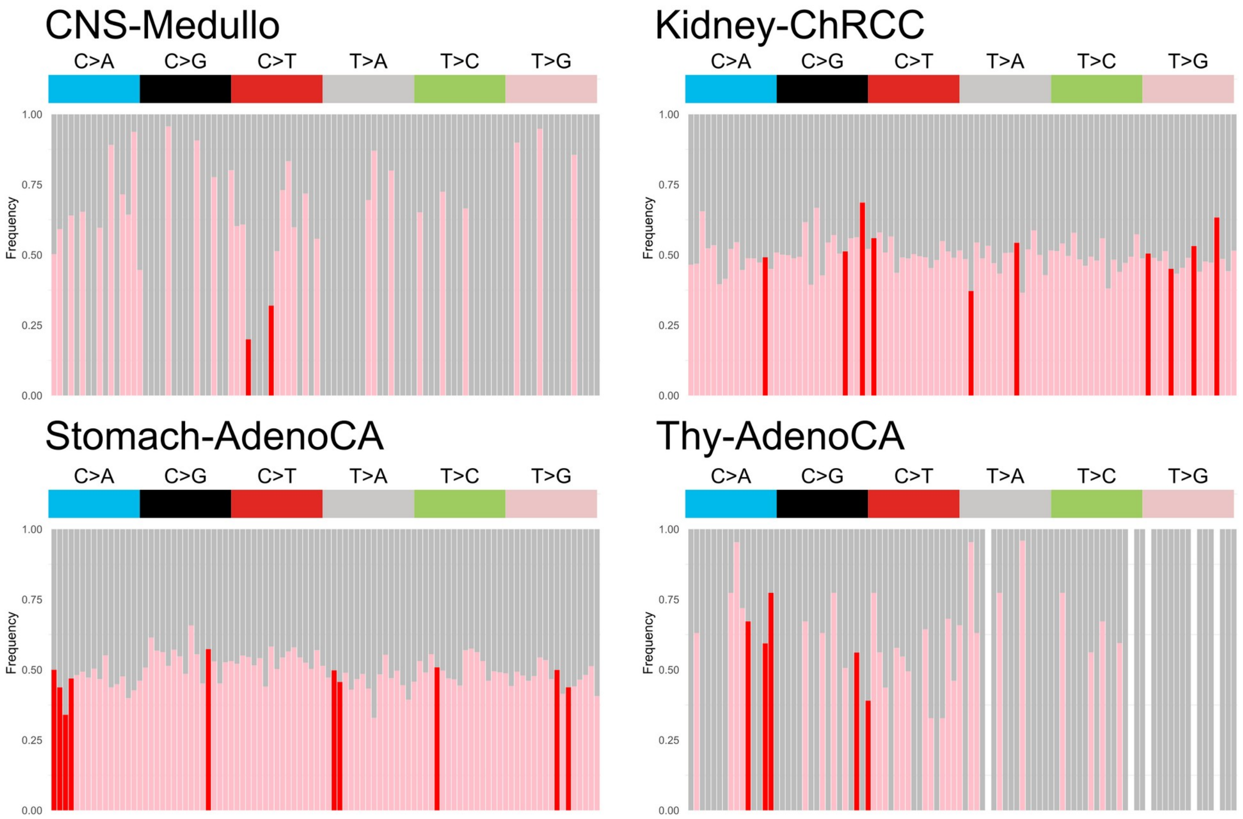

Supplemental Figure S8. The plots show the proportion of specific mutations in marker genes compared to the number of mutations in nonmarker genes for the cancer types that reached 80% accuracy using genes but were below 80% accuracy when using the ANN on WGS_MS+Bins. The colored bars indicate marker gene mutations, while the grey bars symbolise the mutations not in marker genes. The red color indicates that the specific mutation type is part of the top 10 most important mutation types based on the LRP, while the pink indicates that it is not among the top 10.

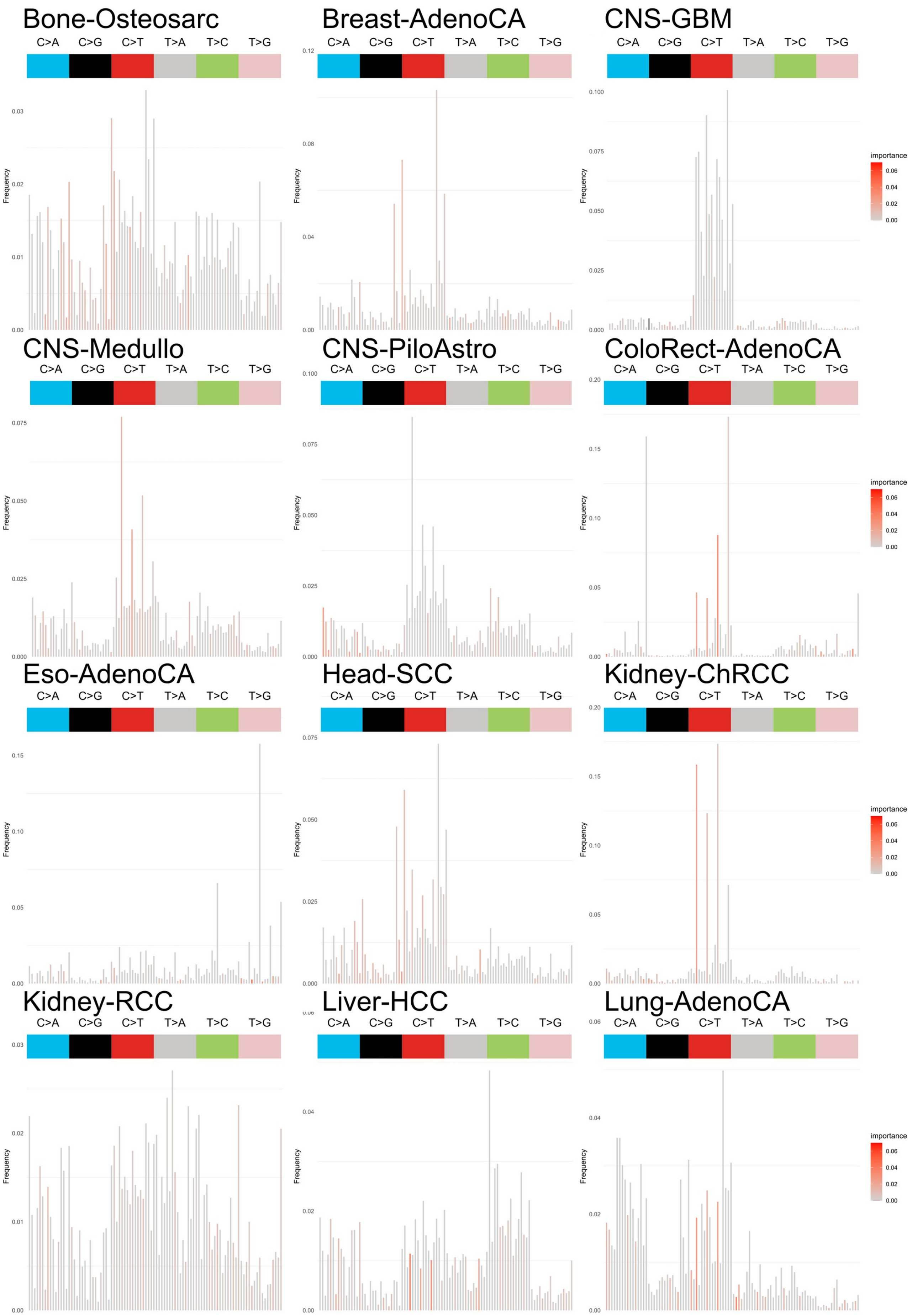

Supplemental Figure S9. Informative mutational signatures, the bars show the mean mutational profile, while the coloring highlights the positive relevance scores of the mutation types.

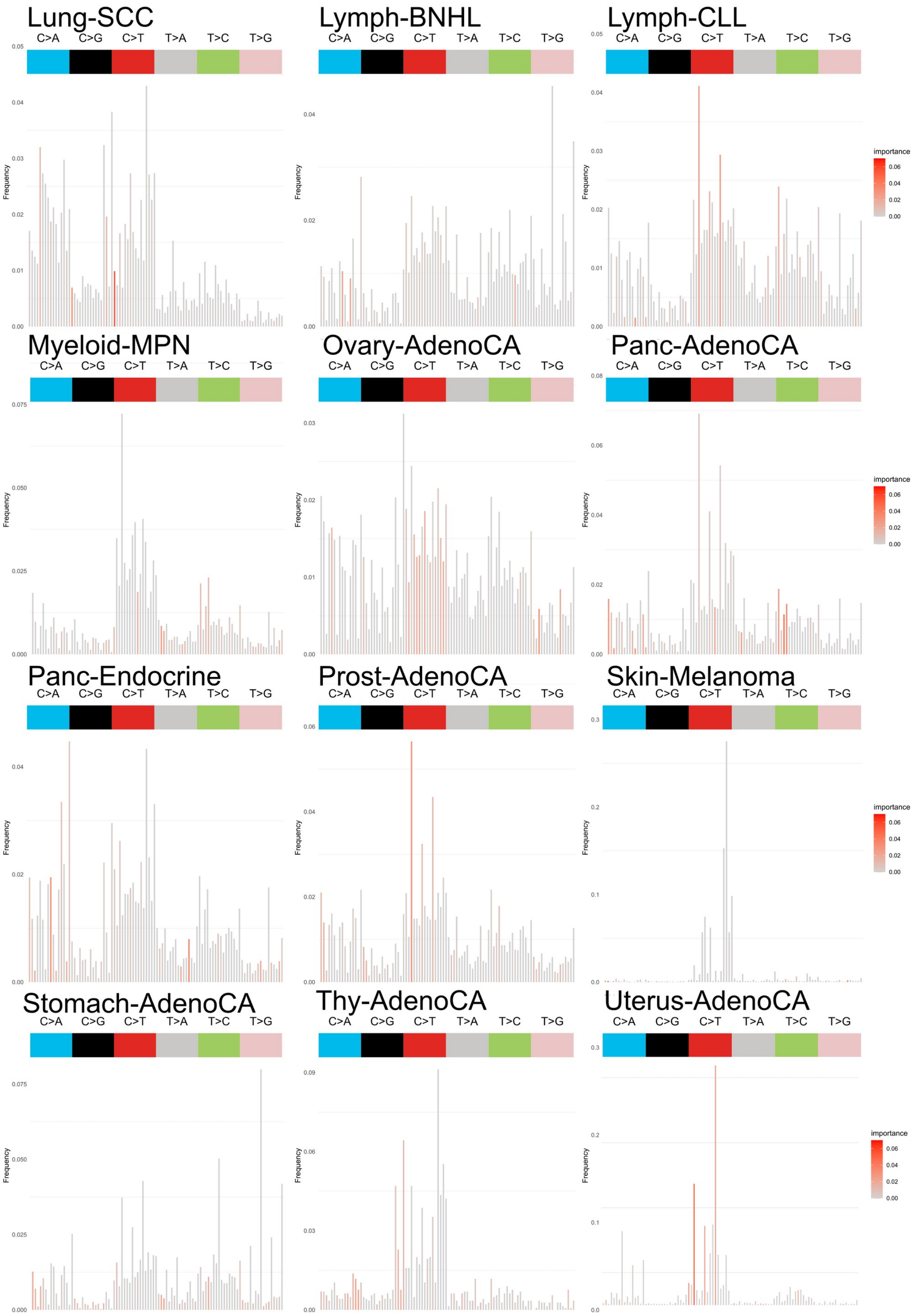

Supplemental Figure S10. Informative mutational signatures, the bars show the mean mutational profile, while the coloring highlights the positive relevance scores of the mutation types.
